# Supplementary material for: Methane-hydrogen-rich fluid migration may trigger seismic failure in subduction zones at forearc depths
Source: Nat Commun. 2024 Jan 11;15:480. doi: 10.1038/s41467-023-44641-w (PMC10784519; doi:10.1038/s41467-023-44641-w)
Supplement: Supplementary file 1 — Supplementary Information [file 41467_2023_44641_MOESM1_ESM.pdf]

Supplementary information

Methane-hydrogen-rich fluid migration may trigger seismic failure in subduction zones at forearc depths

Francesco Giuntoli[1]*, Luca Menegon[2], Guillaume Siron[1], Flavio Cognigni[3], Hugues Leroux[4], Roberto Compagnoni[5], Marco Rossi[3], Alberto Vitale Brovarone,[1,6,7*]

Affiliations

[1]Department of Biological, Geological, and Environmental Sciences, Università degli Studi di Bologna, Bologna, Italy

[2]The Njord Centre, Department of Geosciences, University of Oslo, P.O. Box 1048 Blindern, Norway

[3]Department of basic and applied sciences for engineering (SBAI), Università degli Studi di Roma La Sapienza

[4]Univ. Lille, CNRS, INRAE, Centrale Lille, UMR 8207, UMET, Unité Matériaux et Transformations, F-59000 Lille, France

[5]Dipartimento di Scienze della Terra, Università degli Studi di Torino, via Valperga Caluso 35, 10100 Torino, Italy

[6]Sorbonne Université, Muséum National d'Histoire Naturelle, UMR CNRS 7590, IRD, Institut de Minéralogie, de Physique des Matériaux et de Cosmochimie, IMPMC, 75005 Paris, France

[7]Institute of Geosciences and Earth Resources, National Research Council of Italy, Pisa, Italy

***francesco.giuntoli@unibo.it , *alberto.vitaleb@unibo.it**

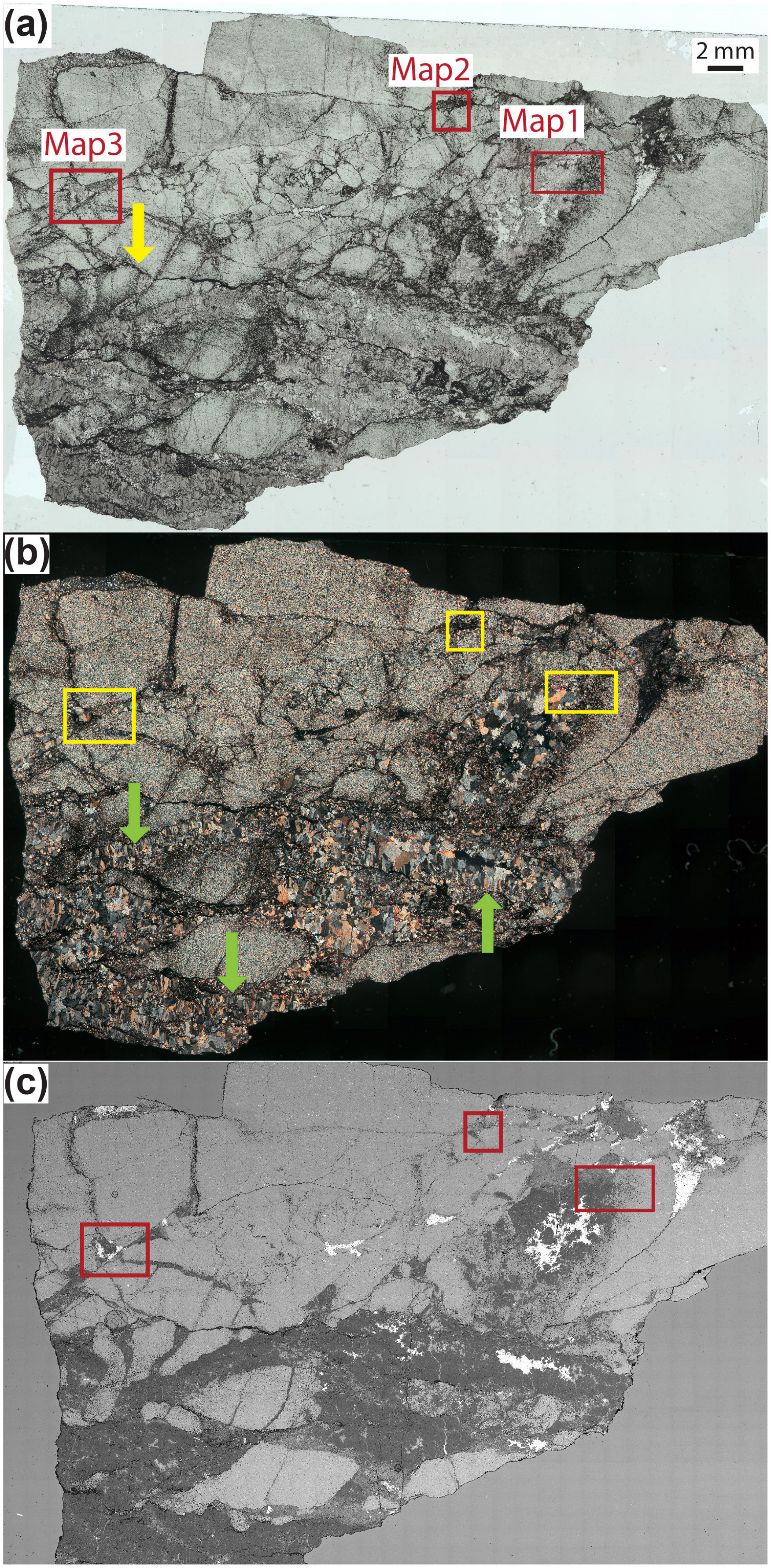

(a)
2 mm
Map2
Map1
Map3
(b)
(c)

Supplementary Figure 1. Thin section scan of brecciated omphacitite with X-ray map locations. The dilation breccia shows predominantly a granular equant growth of jadeite in the matrix, with locally syntaxial growth (green arrows). Compenetrated clasts show graphite-enriched stylolitic structures (yellow arrow). Garnet seems to grow in the innermost part of the dilational matrix. (a) Plane- (b) crossed polarized light and (c) BSE image.

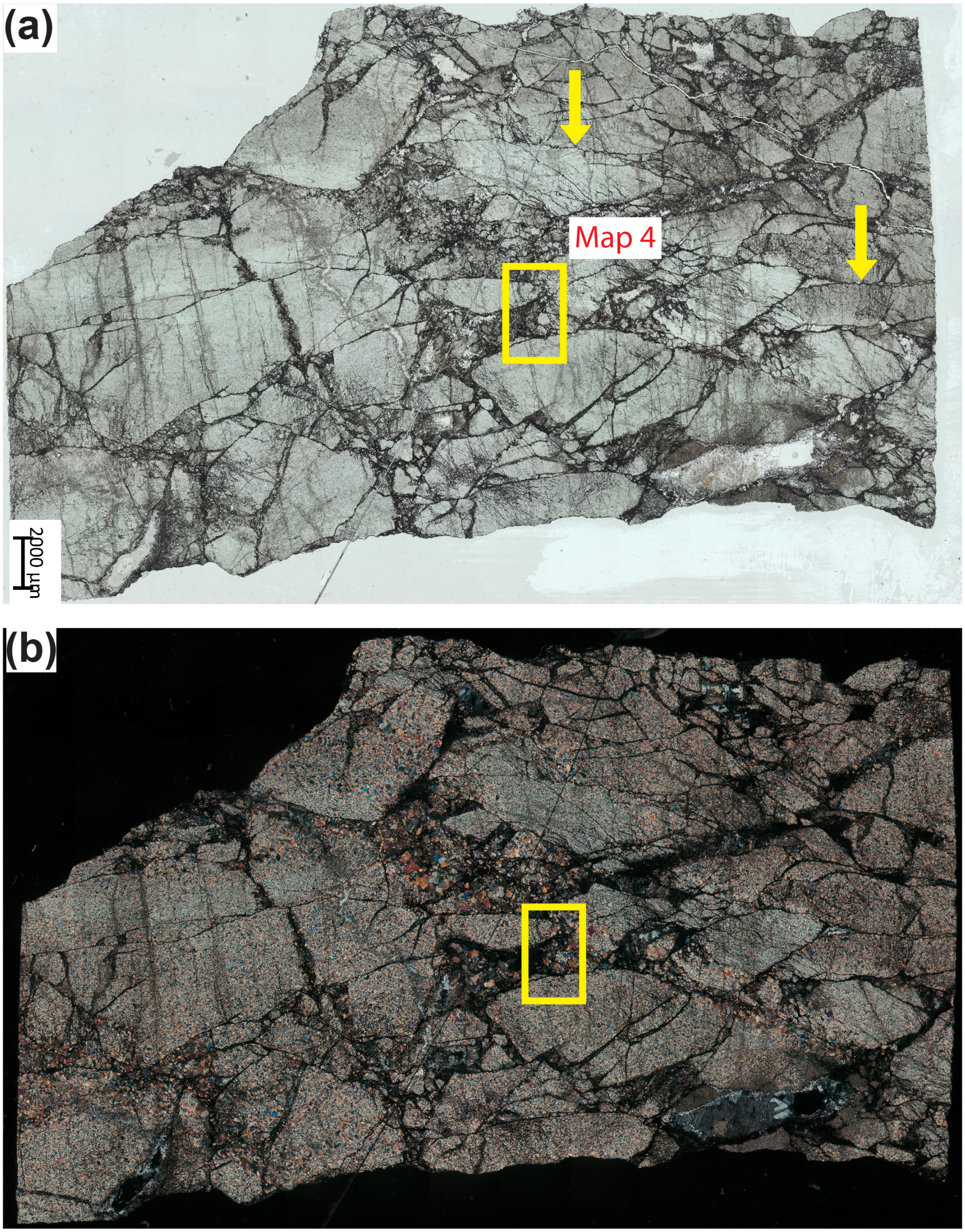

Supplementary Figure 2. (a) Thin section scan of mosaic brecciated omphacitite with X-ray map 4 location (see Fig. S12). Yellow arrows highlight graphite-enriched stylolitic structures. (a) Plane- (b) crossed polarized light.

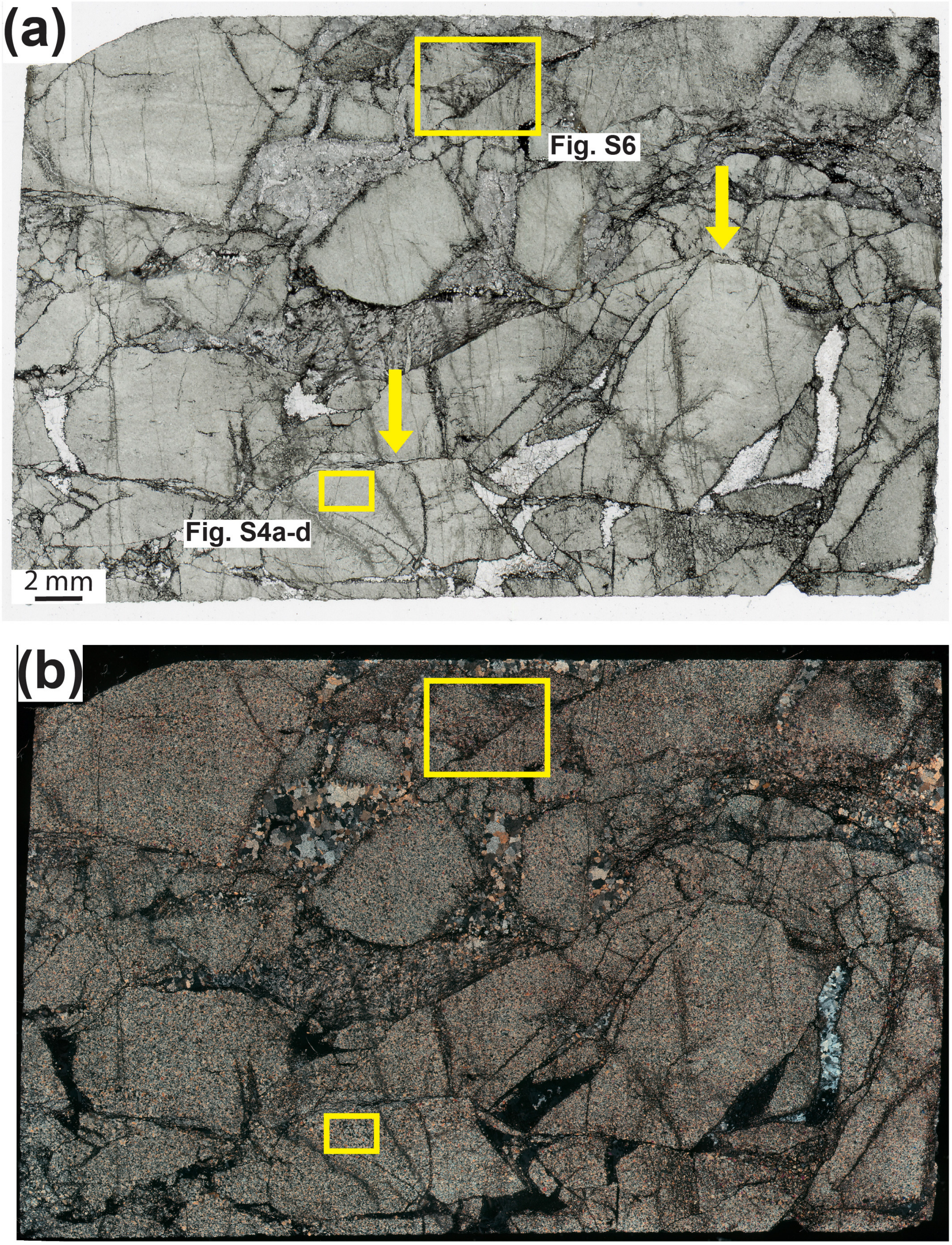

Supplementary Figure 3. Thin section scan of another brecciated omphacitite. The matrix is composed almost exclusively of garnet in the bottom part (white in (a)) and of dendritic/spinifex-like

intergrown of omphacite and jadeite (upper yellow rectangle, see detail in Fig. S6). Compenetrated clasts show graphite-enriched stylolitic structures, as in previous samples (yellow arrow). (a) Plane- (b) crossed polarized light

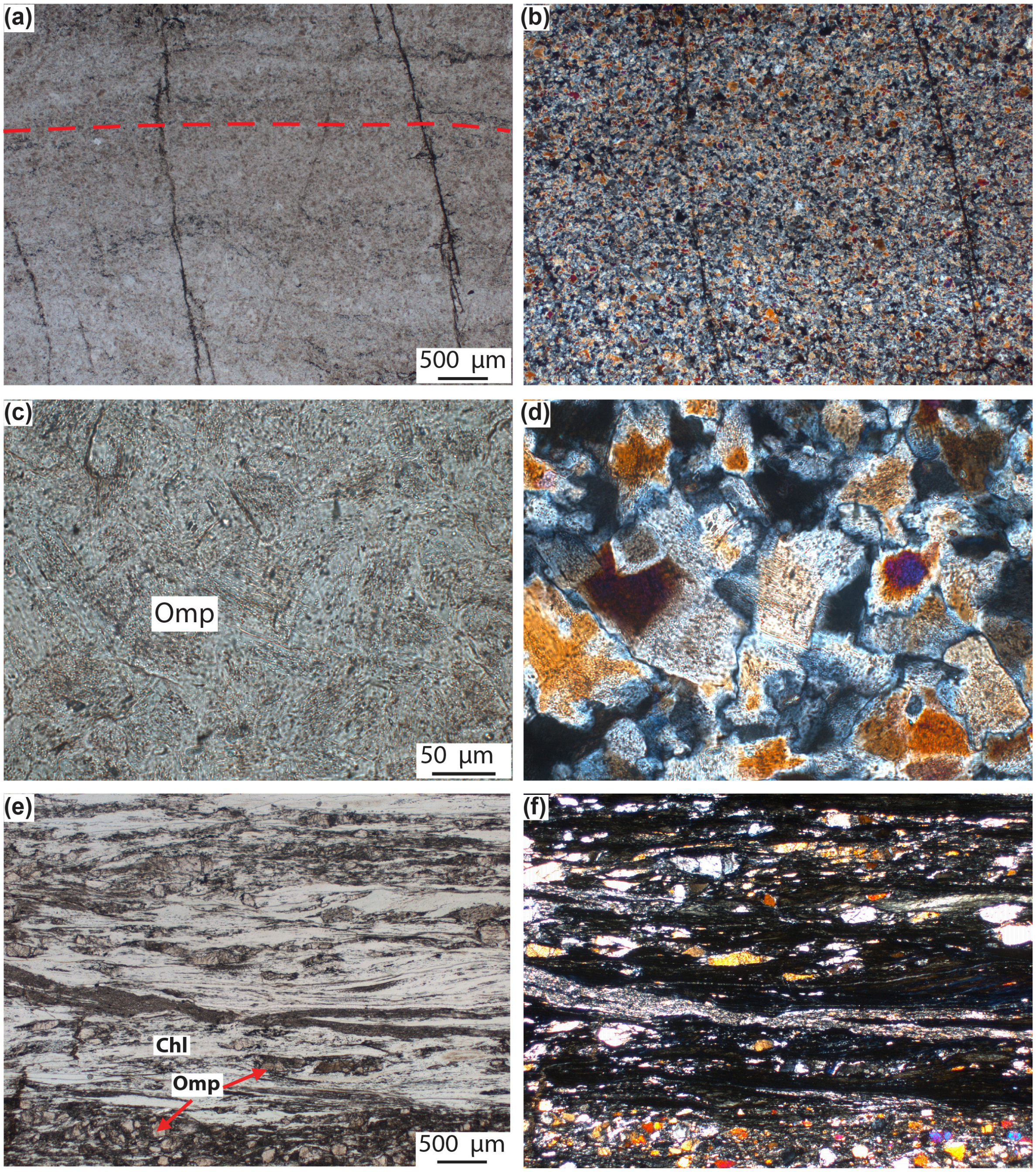

Supplementary Figure 4. (a,b) Thin section images details of Supplementary Fig. 3 showing a weak foliation visible within the omphacitite clasts (red dashed line) and (c,d) detail of the omphacite grains. (e,f) Omphacitite breccia overprinted by a mylonitic foliation rich in chlorite. Plane- (left column) and crossed polarized light (right).

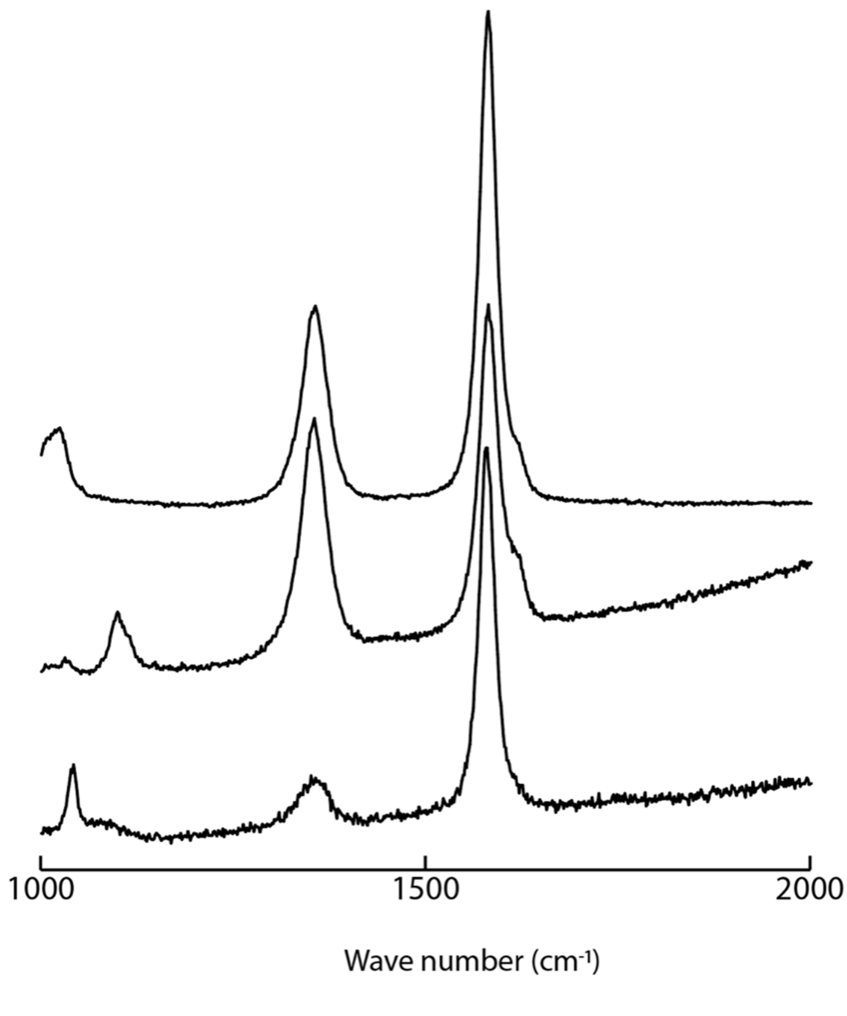

Supplementary Figure 5. Graphite Raman spectra were collected different structural sites, i.e. in vein, along omphacitite clast edges, and as isolated crystals in the breccia matrix. The spectra show some heterogeneity and are consistent with graphitic carbon.

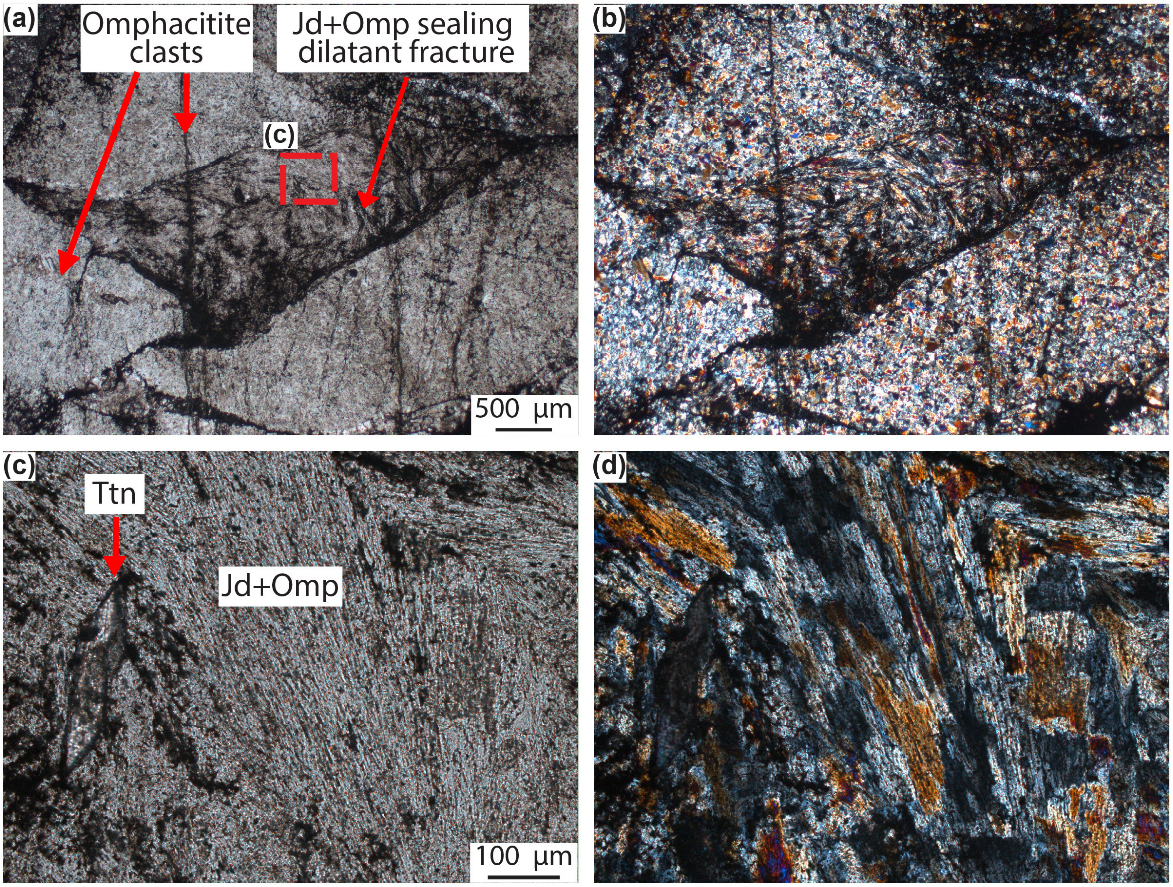

Supplementary Figure 6. (a,b) Thin section images details of Supplementary Fig. 3 displaying dilation breccia with a matrix composed of dendritic/spinifex-like intergrown of omphacite and jadeite. (c,d) Detail of (a,b).

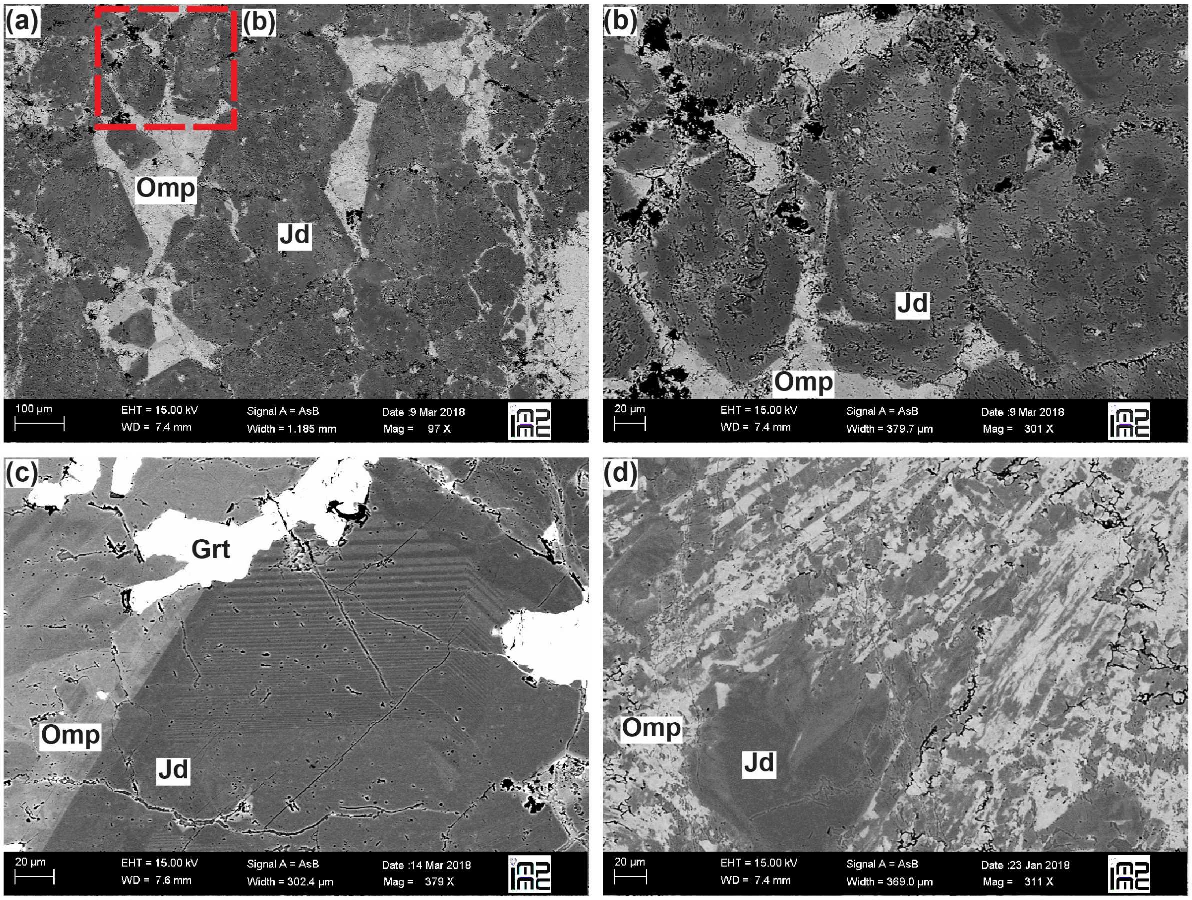

Supplementary Figure 7. BSE images of (a,b) zoned jadeite and omphacite growing in the matrix, (c) oscillatory zoning of jadeite and (d) dendritic textures of omphacite (brighter) and jadeite (darker).

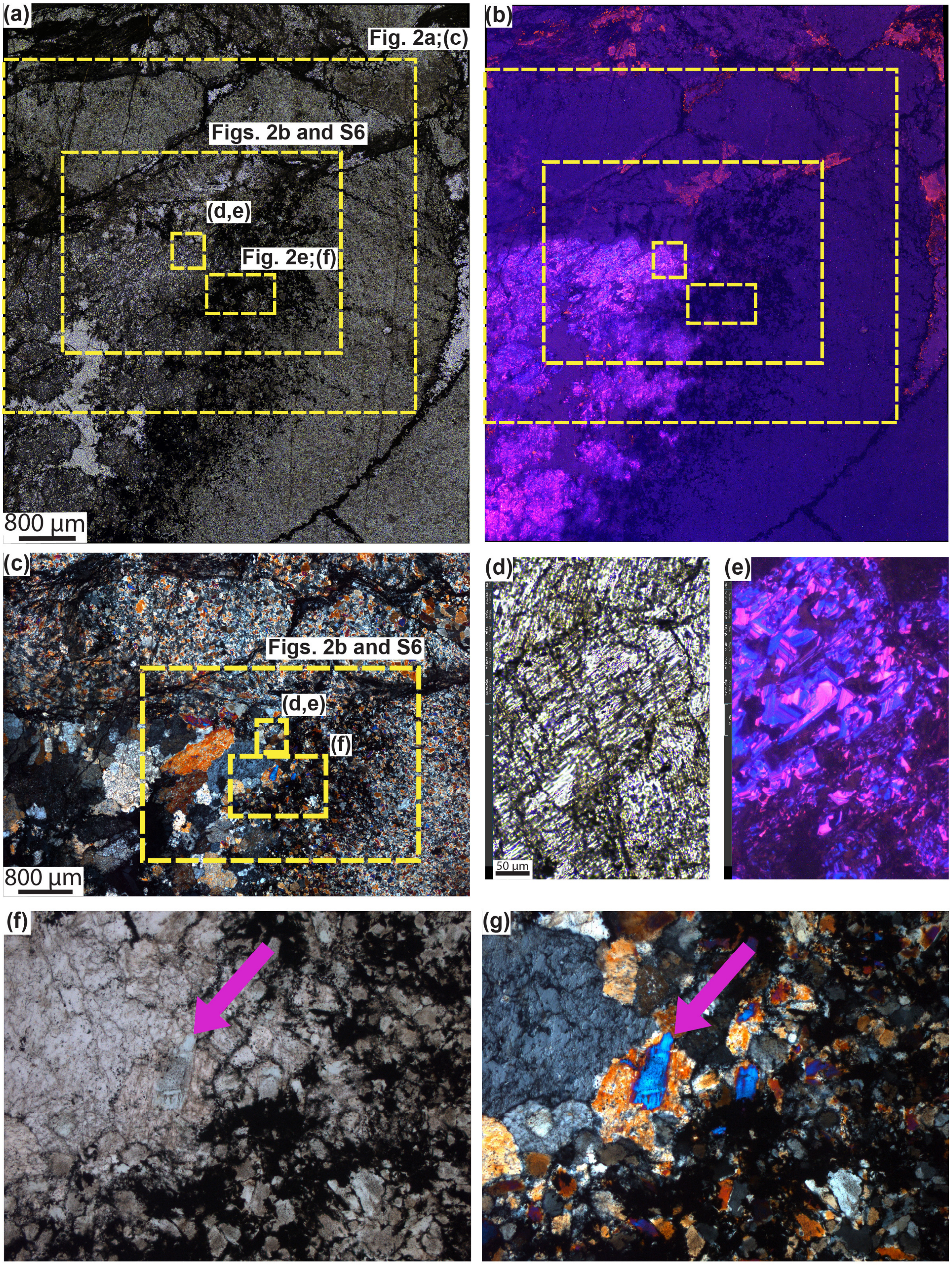

Supplementary Figure 8. (a) Detail of the contact between omphacitite clasts and matrix, plane-polarized light. (b) CL image highlighting jadeite grains located in the matrix with pink and light blue colours, and garnet with orange and blue colours. (c) Detail of (a) and same area of Fig. 2a, crossed-polarized light. Note the different grain size between smaller omphacite grains in the clasts and larger

jadeite grains in the matrix. (d,e) Detail of jadeite grains displaying oscillatory growth zoning, plane-polarized light and CL image, respectively. (f,g) Detail of (c). The arrow highlights a grain of omphacite generation 1 and 2 overgrown by jadeite (see text for discussion and compare with Supplementary Fig. 6b and c).

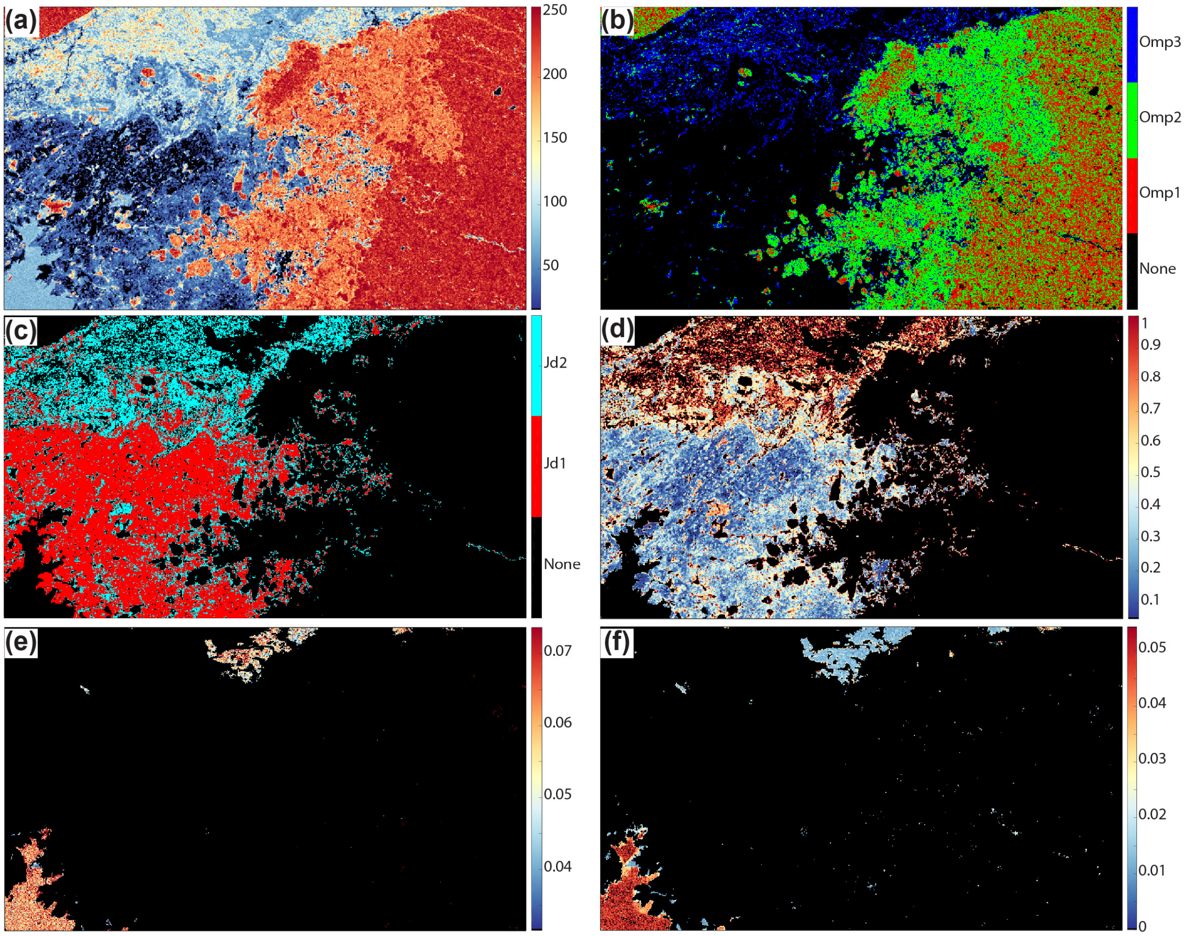

Supplementary Figure 9. Complementary insets of standardized X-ray map 1 shown in Fig. 2d (see location in Supplementary Fig. 1). (a) Fe counts per second highlighting the relations between omphacite, jadeite and garnet. (b, c) Classification of jadeite and omphacite generations, respectively (see text for explanation). (d) FeO weight % in jadeite. (e, f) Garnet X_{Grs} and X_{Adr} maps, respectively.

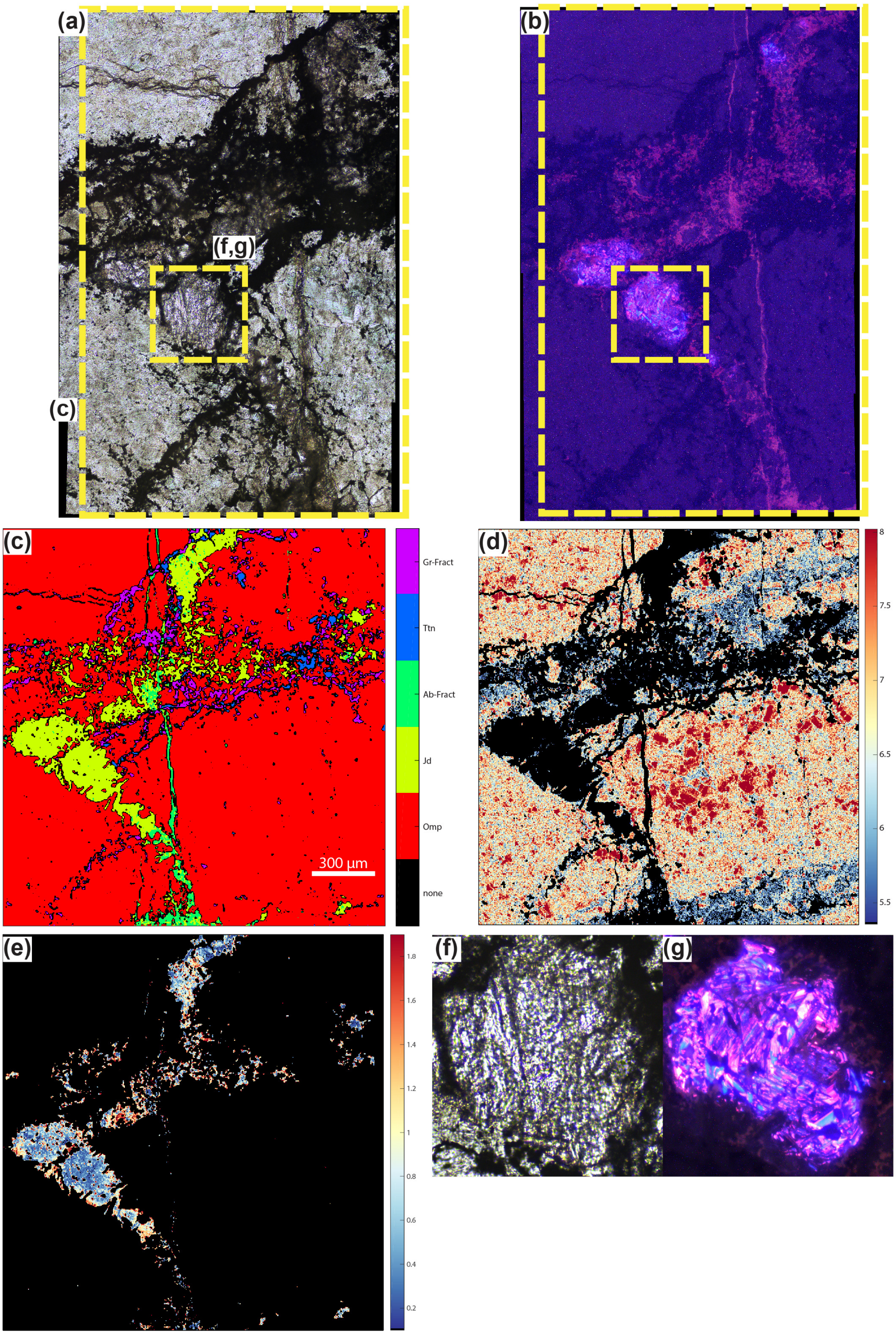

(a)
(f,g)
(c)
(b)
(c)
Gr-Fract
Ttn
Ab-Fract
Jd
Omp
none
300 µm
(d)
8
7.5
7
6.5
6
5.5
(e)
1.8
1.6
1.4
1.2
1
0.8
0.6
0.4
0.2
(f)
(g)

Supplementary Figure 10. X-ray Map 2 (see location in Supplementary Fig. 1). (a,b) Plane-polarized light and CL image, respectively. X-ray map 2: (c) colour coded for the different mineral phases. Gr-fract: graphite-coated fractures, Ttn: titanite, Ab-Fract: later albite fracture, Jd: jadeite, Omp: Omphacite. (d,e) FeO weight % in omphacite and jadeite, respectively. (f,g) Detail of jadeite in the matrix; plane- polarized light and CL image, respectively.

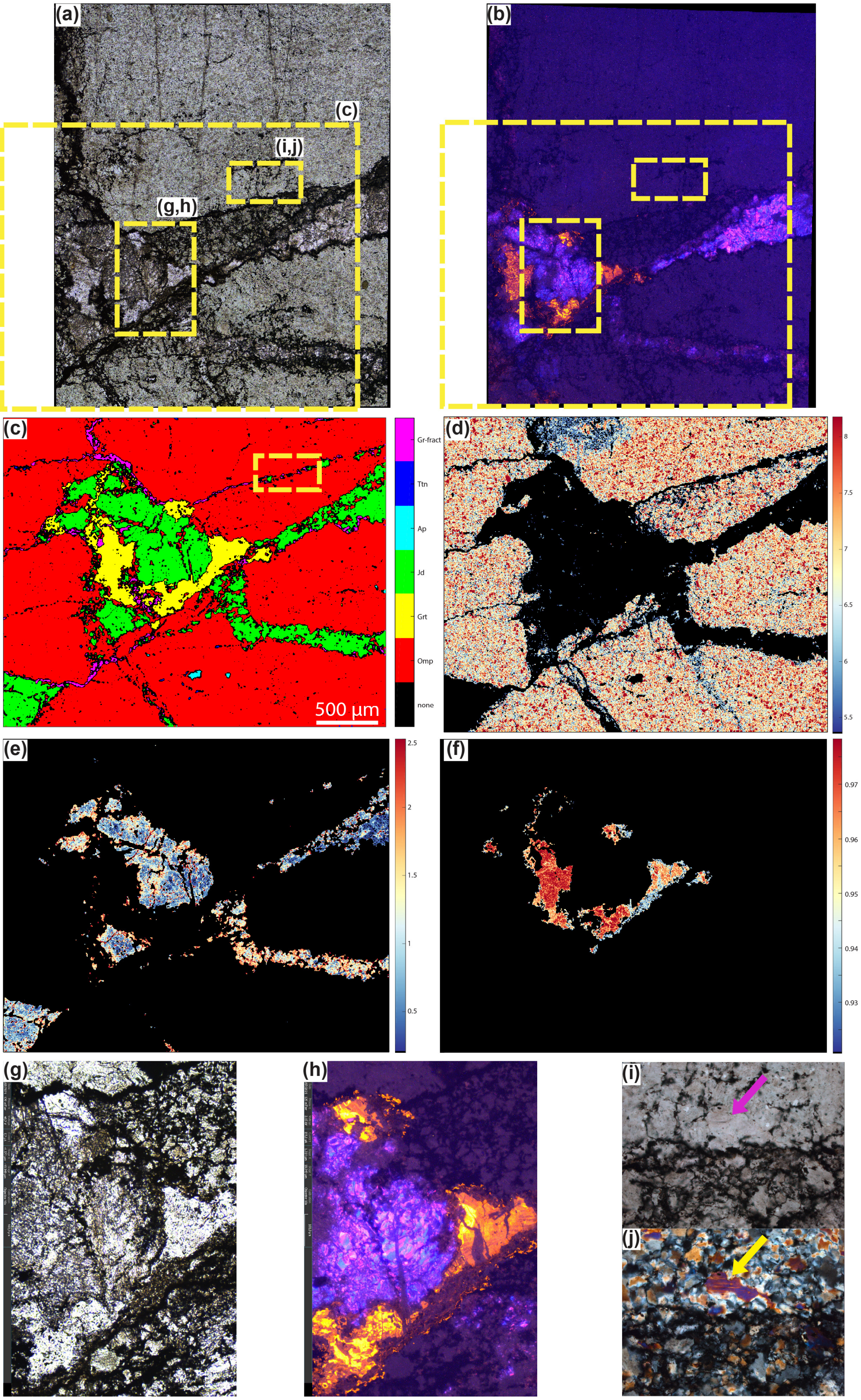
(a)
(c)
(i,j)
(g,h)
(b)
(c)
Gr-fract
Ttn
Ap
Jd
Grt
Omp
none
500 µm
(d)
8
7.5
7
6.5
6
5.5
(e)
2.5
2
1.5
1
0.5
(f)
0.97
0.96
0.95
0.94
0.93
(g)
(h)
(i)
(j)

Supplementary Figure 11. Detail of the contact between omphacitite clasts and matrix and X-ray Map 3 (see location in Supplementary Fig. 1). (a,b) Plane- polarized light and CL image, respectively. (c-f) X-ray map 3: (c) colour coded for the different mineral phases. Gr-fract: graphite-coated fractures, Ttn: titanite, Ap: apatite, Jd: jadeite, Grt: garnet, Omp: Omphacite. (d,e) FeO weight % in omphacite and jadeite, respectively. (f) Garnet X_{Grs}. (g, h) Detail of jadeite and garnet in the matrix; plane- polarized light and CL image, respectively. (i,j) Omphacite grain in the omphacitite clast close to a graphite-coated fracture displaying twinning (see text for discussion).

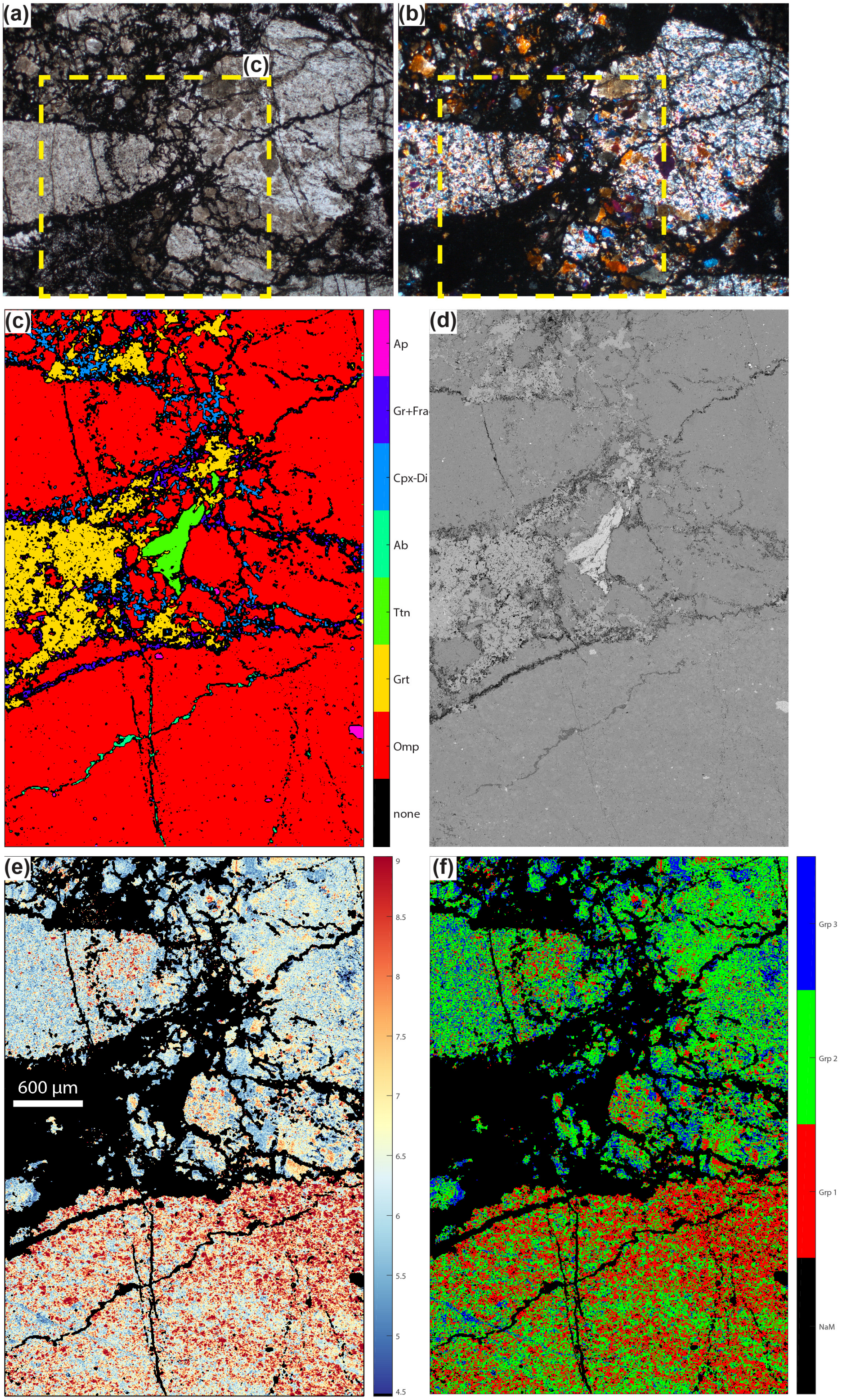
(a)
(b)
(c)
(d)
(e)
(f)
Ap
Gr+Fra
Cpx-Di
Ab
Ttn
Grt
Omp
none
600 µm
9
8.5
8
7.5
7
6.5
6
5.5
5
4.5
Grp 3
Grp 2
Grp 1
NaM

Supplementary Figure 12. X-ray Map 4 (see location in Supplementary Fig. 2). (a,b) Detail of the contact between two omphacitite clasts. Plane and crossed-polarized light, respectively. (c,f) X-ray map 4: (c) colour coded for the different mineral phases. Ap: Apatite, Gr-fra: graphite-coated fractures, Cpx-Di: retrograde Diopside-rich clinopyroxene, Ttn: titanite, Ab-Fract: later albite fracture, Omp: Omphacite. (d) BSE image. (e) FeO weight % in omphacite. (f) Classification of omphacite generations (see text for explanation).

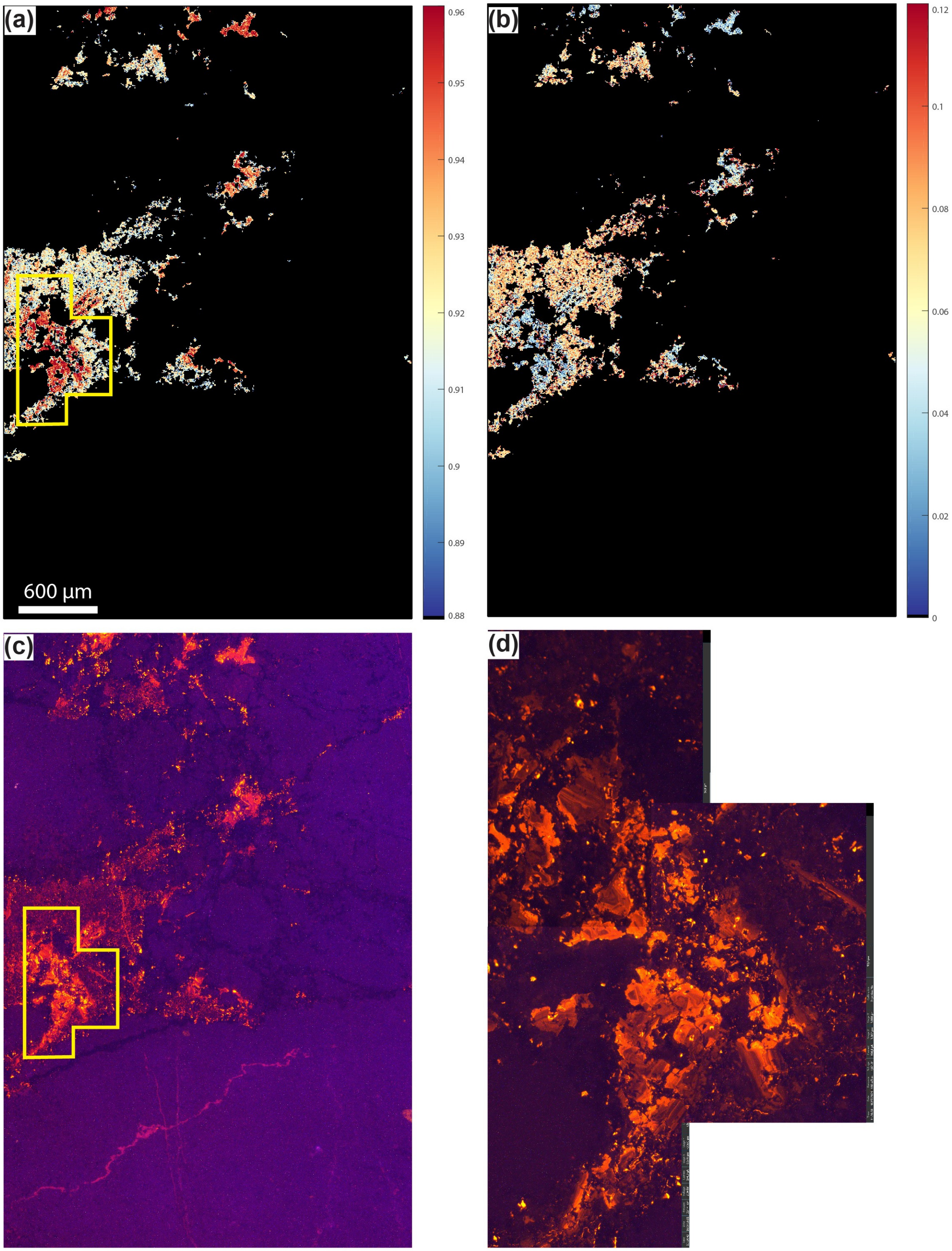

Supplementary Figure 13. Continuation of X-ray Map 4 (see location in Supplementary Fig. 2). (a) Garnet X_{Grs}, (b) X_{Adr}, and (c, d) CL images highlighting a core to rim zoning.

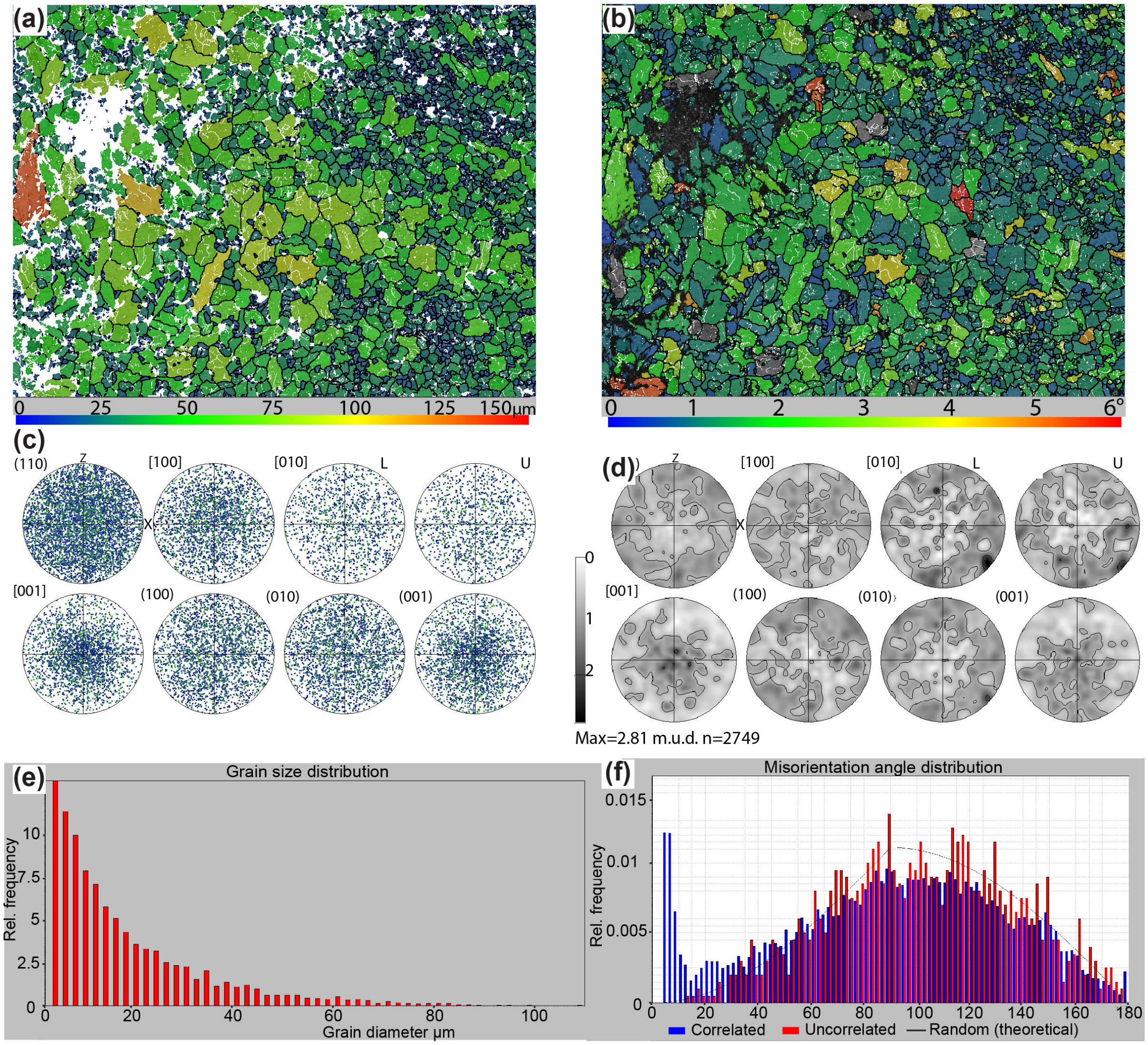

Supplementary Figure 14. Complementary insets of EBSD map of omphacite grains located inside the clast (compare with Fig. 2,f). (a) Grain size map. (b) Grain orientation spread (GOS) map; the grains show numerous low-angle boundaries and relative low GOS values except a few grains, with values up to 6°. (c,d) Pole figures and contoured pole figures of the crystallographic orientation data of omphacite showing that no CPO is present. 2749 data points (one-point-per-grain). n = number of grains. Half width 10° and cluster size 5°, maximum value is given. Lower hemisphere of the stereographic projection, if not specified. (e, f) Histogram of grain size and misorientation angle distribution, respectively.

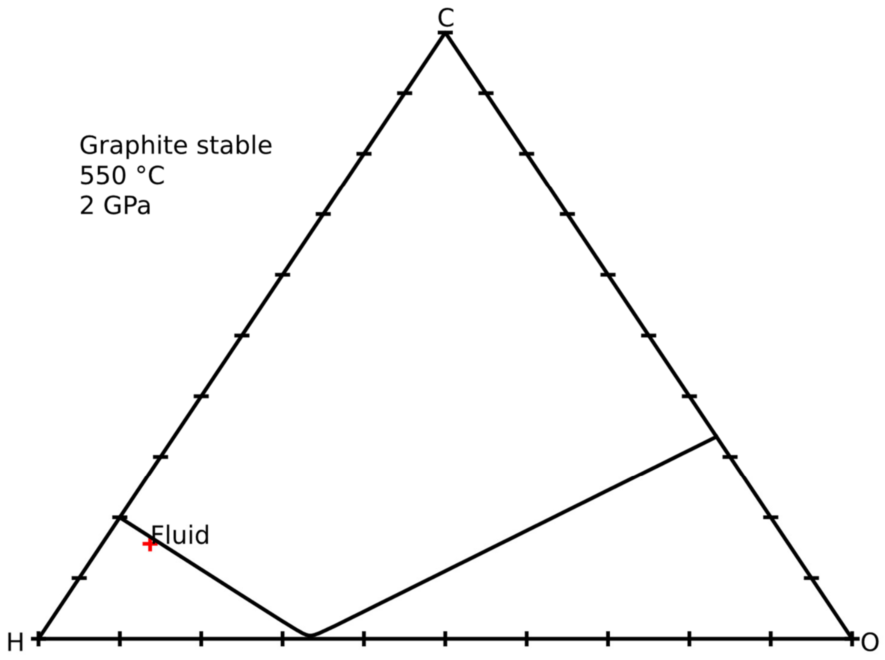

Supplementary Figure 15. C-O-H ternary diagram showing the composition of a fluid having the CH_4/H_2 proportion as derived from Raman analysis of the gaseous part of fluid inclusions included in jadeite forming the breccia matrix. The position of the fluid appears slightly below the carbon saturation curve, and correspond to an X_O value of 0.07. See Methods for details.

X-ray Microscopy (XRM) statistical data

#1_4a-2

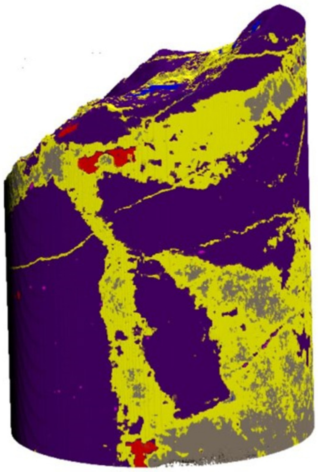

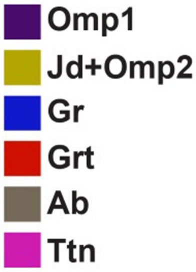

Mineral	Value
Titanite	0.11%
Omphacite 1	44.08%
Garnet	1.76%
Graphite	1.34%
Omphacite 2 & Jadeite	30.06%
Albite	22.76%
Sum	100.11%

#1_4a-2 – Volume Titanite

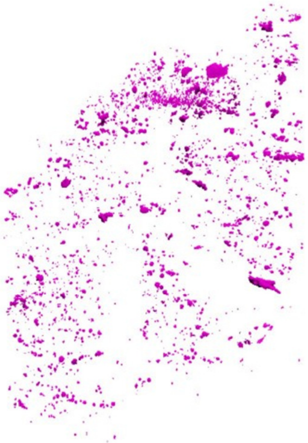

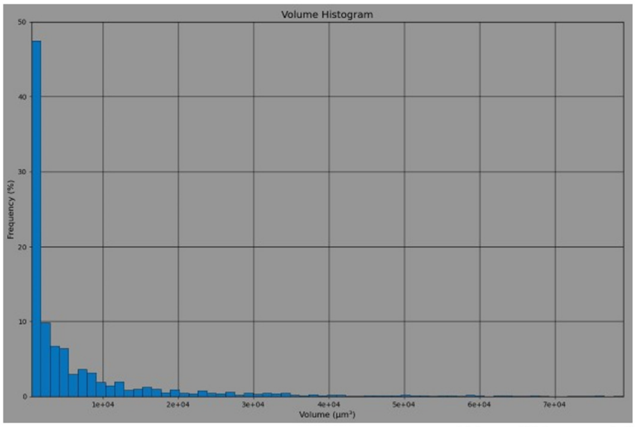

Volume Histogram statistics

- Min: 526.74 µm³
- Max: 3,955,823.76 µm³
- Mean: 12,894.62 µm³
- Standard dev: 84,268.18 µm³

#1_4a-2 – Mean Feret Diameter
Titanite

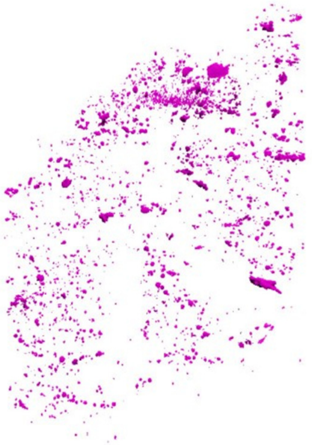

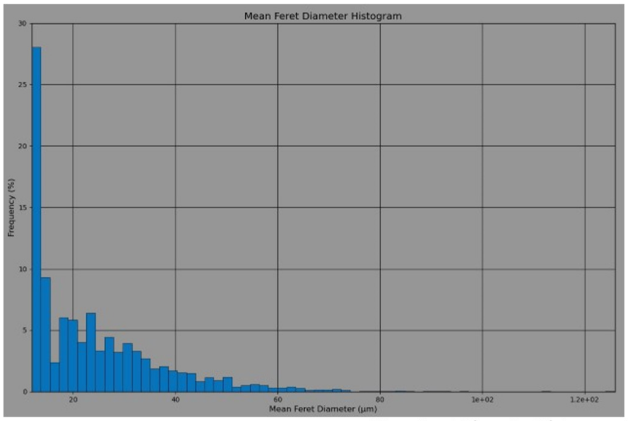

Mean Feret Diameter Histogram statistics
- Min: 11.90 µm
- Max: 303.91 µm
- Mean: 26.10 µm
- Standard dev: 19.90 µm

#1_4a-2 – Volume
Omphacite 1

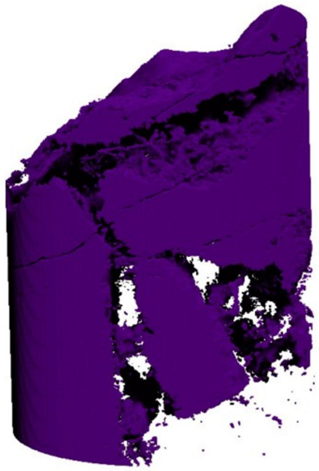

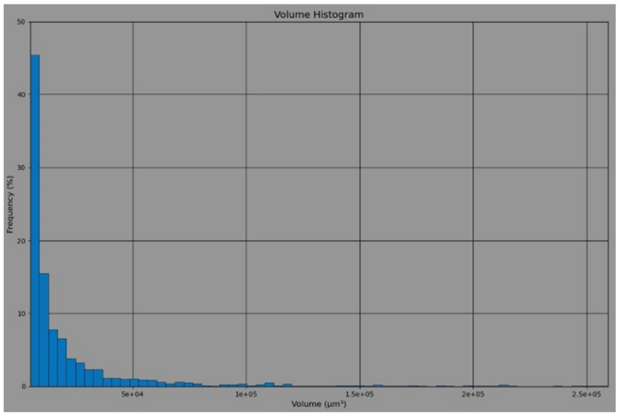

Volume Histogram statistics
- Min: 4,740.67 µm³
- Max: 17,400,603,631.49 µm³
- Mean: 6,463,548.46 µm³
- Standard dev: 333,152,386.87 µm³

#1_4a-2 – Mean Feret Diameter
Omphacite 1

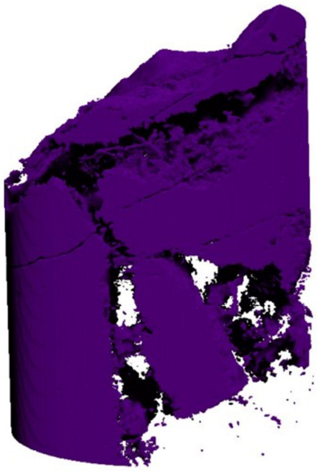

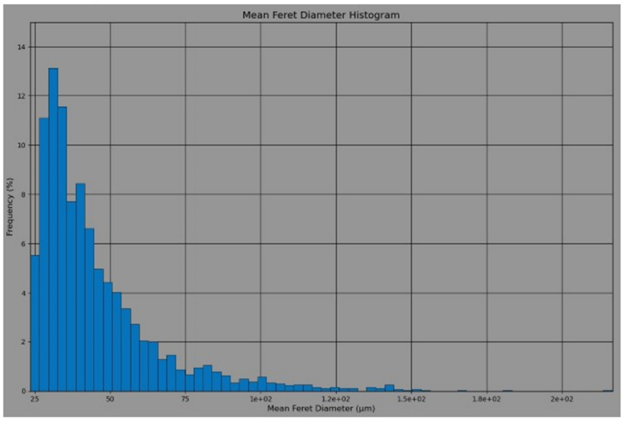

Mean Feret Diameter Histogram statistics

- Min: 23.47 µm
- Max: 4,307.83 µm
- Mean: 52.19 µm
- Standard dev: 93.51 µm

#1_4a-2 – Volume
Omphacite 2 & Jadeite

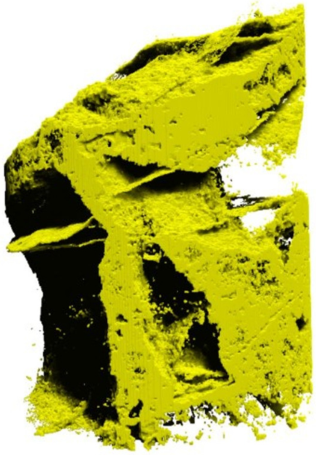

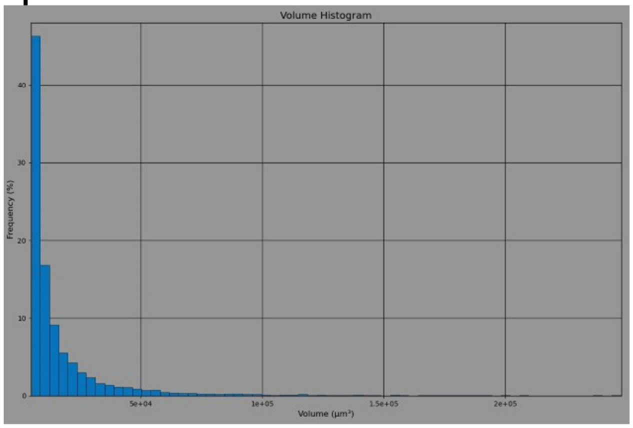

Volume Histogram statistics

- Min: 4,740.67 µm³
- Max: 11,870,067,046.10 µm³
- Mean: 2,406,371.69 µm³
- Standard dev: 168,036,015.74 µm³

#1_4a-2 – Mean Feret Diameter
Omphacite 2 & Jadeite

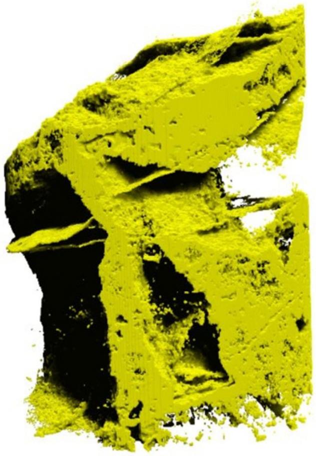

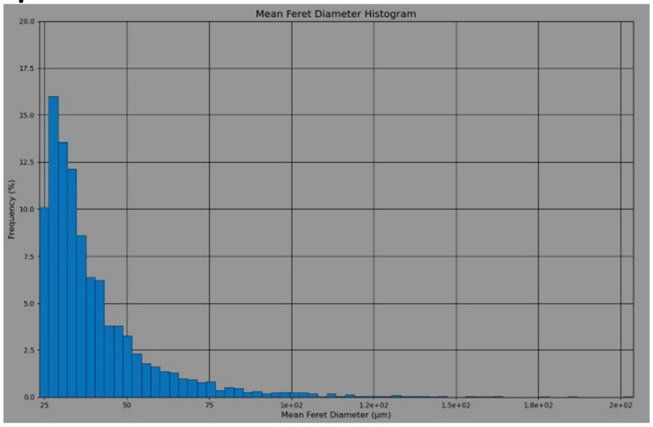

Mean Feret Diameter Histogram statistics

- Min: 23.39 µm
- Max: 4,671.25 µm
- Mean: 42.44 µm
- Standard dev: 70.61 µm

#1_4a-2 – Volume
Garnet

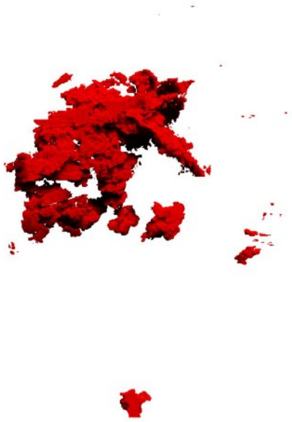

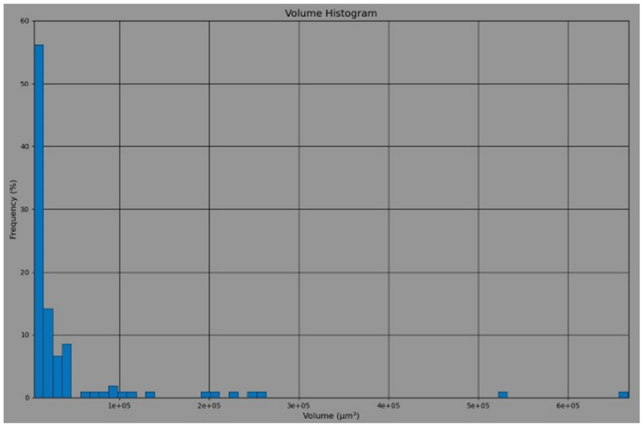

Volume Histogram statistics

- Min: 4,740.67 µm³
- Max: 627,334,125.94 µm³
- Mean: 6,065,461.71 µm³
- Standard dev: 58,000,508.26 µm³

#1_4a-2 – Mean Feret Diameter
Garnet

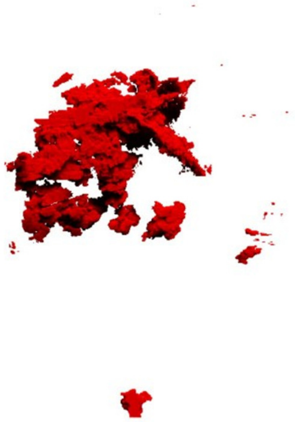

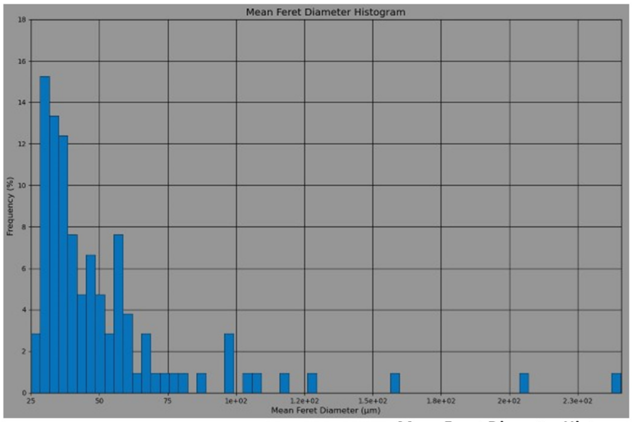

Mean Feret Diameter Histogram statistics
- Min: 24.89 µm
- Max: 2,018.45 µm
- Mean: 94.42 µm
- Standard dev: 201.97 µm

#1_4a-2 – Volume
Graphite

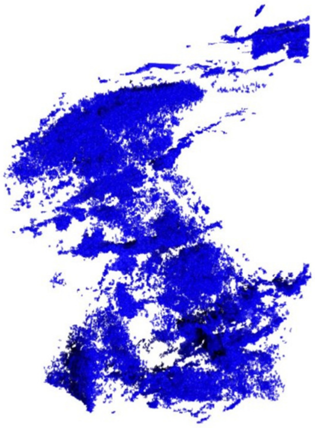

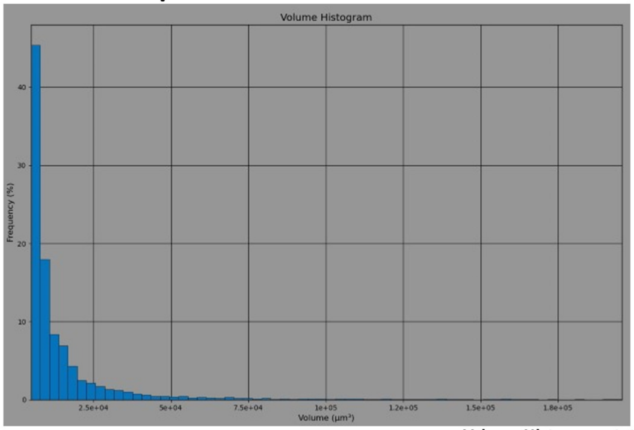

Volume Histogram statistics
- Min: 4,740.67 µm³
- Max: 93,321,095.52 µm³
- Mean: 67,510.61 µm³
- Standard dev: 1,443,868.98 µm³

#1_4a-2 – Mean Feret Diameter
Graphite

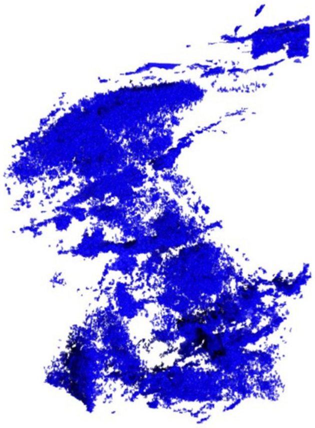

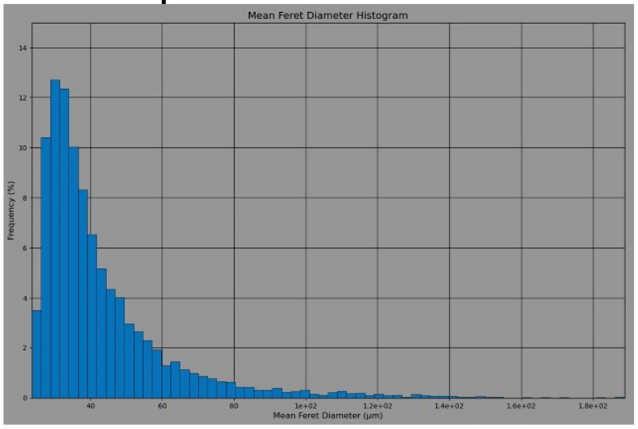

Mean Feret Diameter Histogram statistics
- Min: 23.65 µm
- Max: 1,550.09 µm
- Mean: 47.04 µm
- Standard dev: 47.98 µm

#1_4a-2 – Volume
Albite

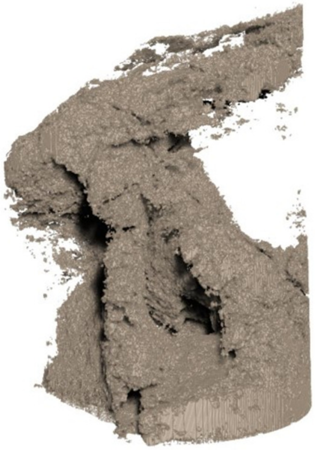

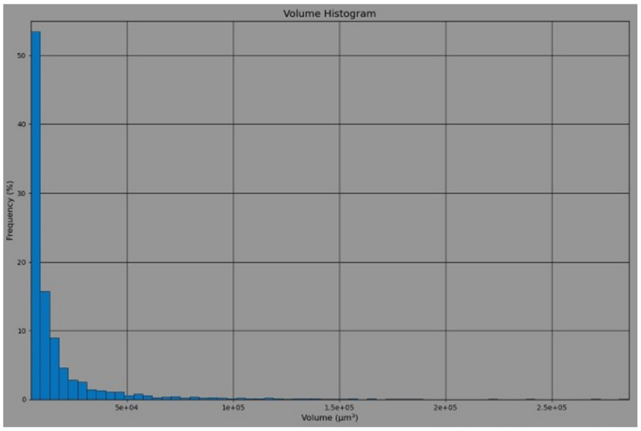

Volume Histogram statistics
- Min: 4,740.67 µm³
- Max: 8,969,916,751.63 µm³
- Mean: 2,370,017.16 µm³
- Standard dev: 144,902,045.25 µm³

#1_4a-2 – Mean Feret Diameter Albite

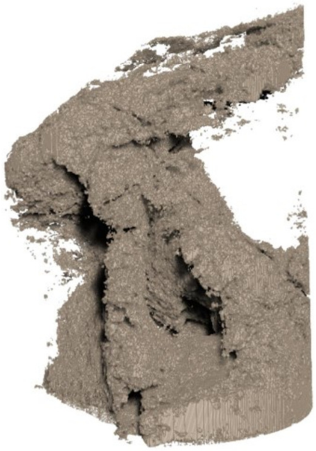

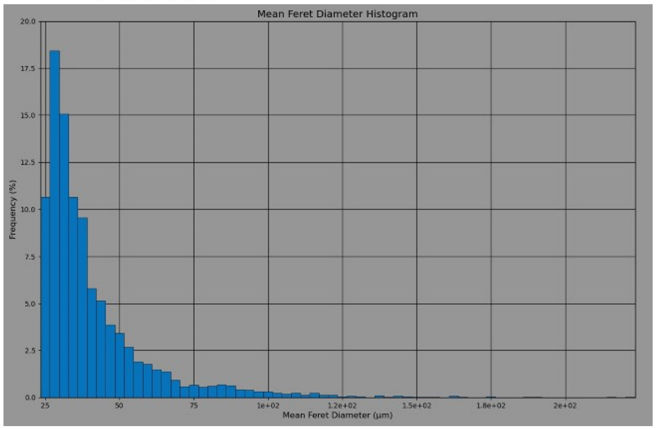

Mean Feret Diameter Histogram statistics

- Min: 23.44 µm
- Max: 4,590.19 µm
- Mean: 44.54 µm
- Standard dev: 80.67 µm

#1_4a-2_ROI 1

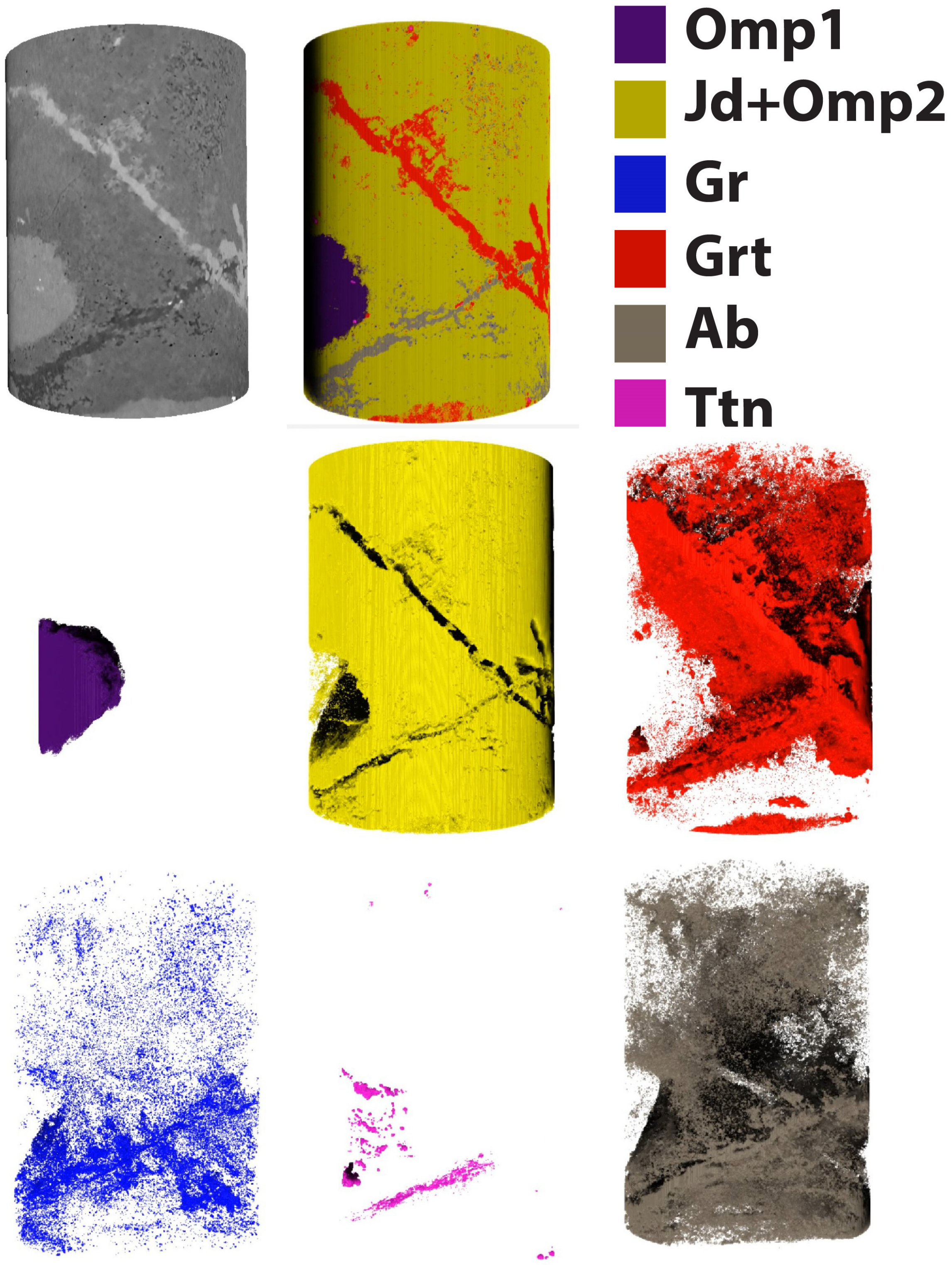

#1_4a-2, ROI 1

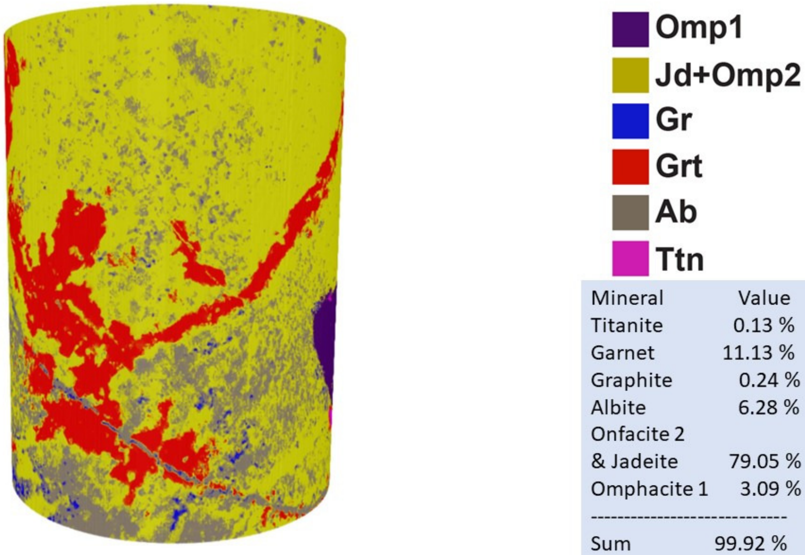

Mineral	Value
Titanite	0.13 %
Garnet	11.13 %
Graphite	0.24 %
Albite	6.28 %
Onfacite 2 & Jadeite	79.05 %
Omphacite 1	3.09 %

Sum	99.92 %

#1_4a-2, ROI 1 – Volume Graphite

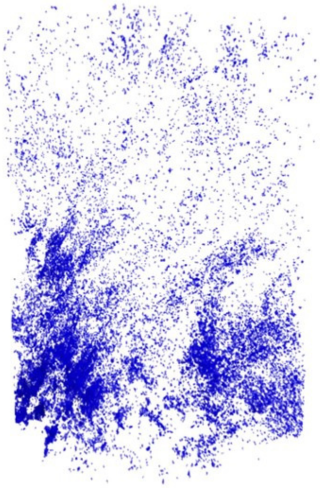

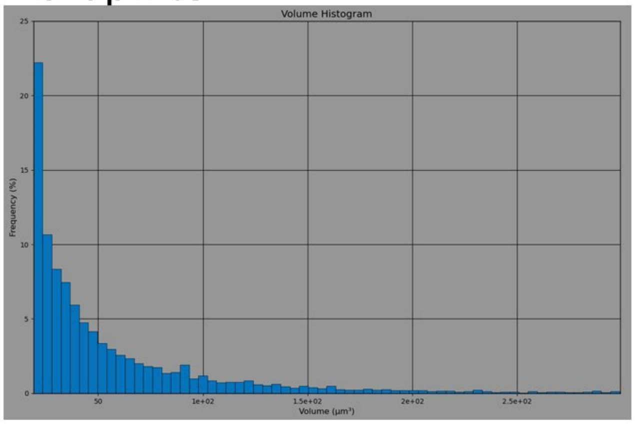

Volume Histogram statistics

- Min: 19.08 µm³
- Max: 96,677.99 µm³
- Mean: 96.11 µm³
- Standard dev: 1,059.12 µm³

#1_4a-2, ROI 1 – Mean Feret Diameter Graphite

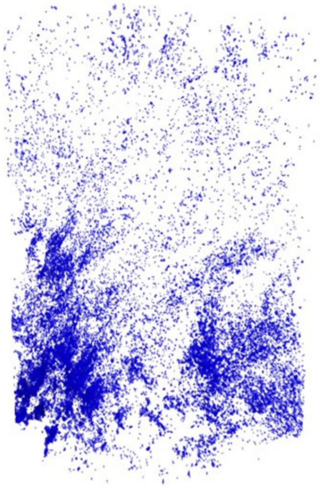

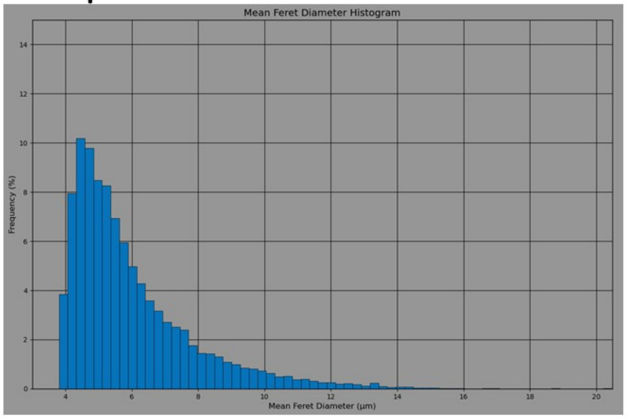

Mean Feret Diameter Histogram statistics

- Min: 3.80 µm
- Max: 138.94 µm
- Mean: 6.42 µm
- Standard dev: 3.66 µm

#1_4a-2, ROI 1 – Mean Feret Diameter Albite

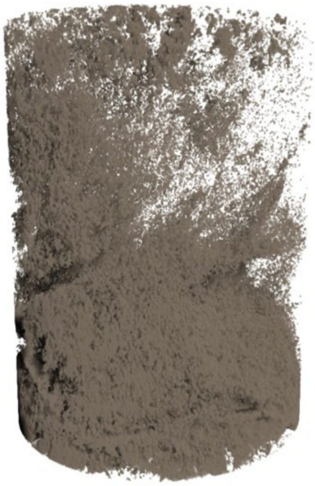

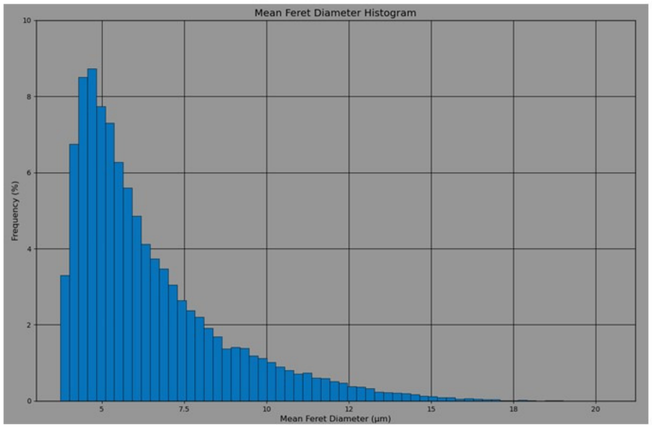

Mean Feret Diameter Histogram statistics

- Min: 3.74 µm
- Max: 927.98 µm
- Mean: 7.24 µm
- Standard dev: 6.14 µm

#1_4a-2, ROI 1 – Volume
Albite

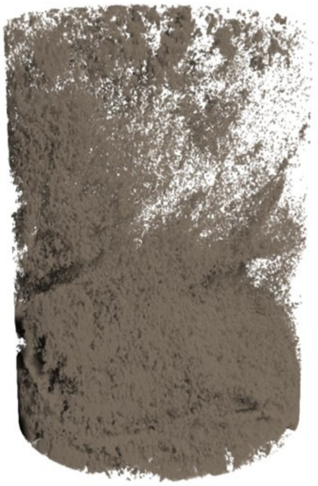

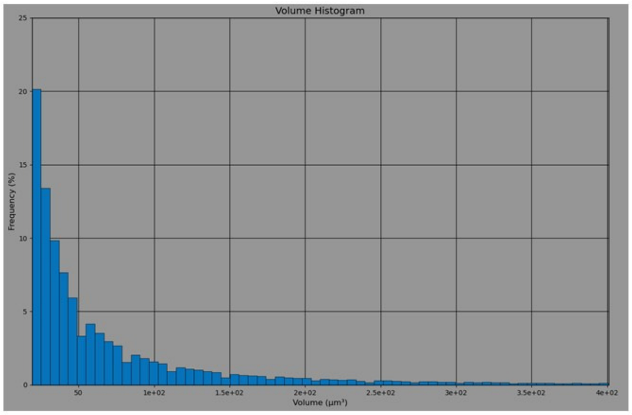

Volume Histogram statistics
- Min: 19.08 µm³
- Max: 28,840,359.87 µm³
- Mean: 602.17 µm³
- Standard dev: 113,422.34 µm³

#1_4a-2, ROI 1 – Mean Feret Diameter
Omphacite 2 & Jadeite

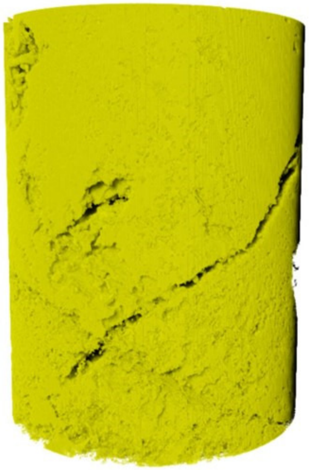

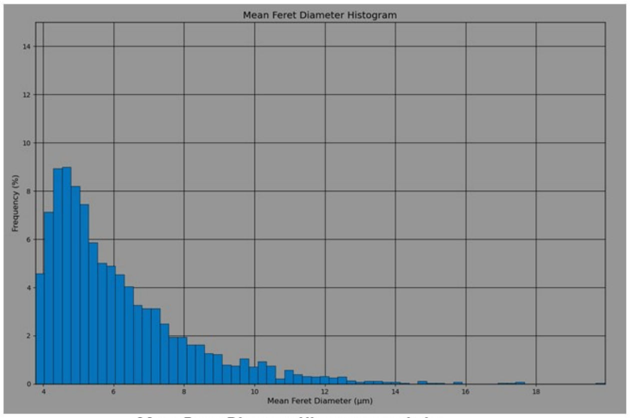

Mean Feret Diameter Histogram statistics
- Min: 3.77 µm
- Max: 1,175.39 µm
- Mean: 7.12 µm
- Standard dev: 21.93 µm

#1_4a-2, ROI 1 – Volume
Omphacite 2 & Jadeite

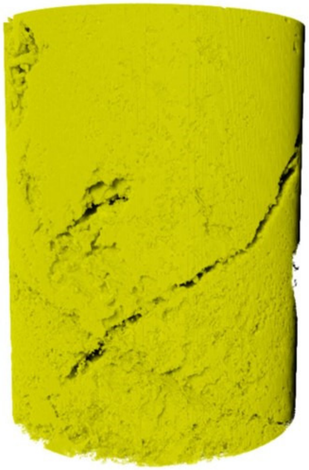

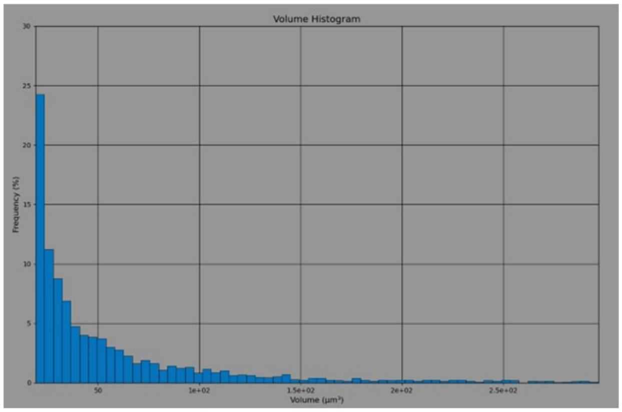

Volume Histogram statistics

- Min: 19.08 µm³
- Max: 502,161,584.68 µm³
- Mean: 171,786.38 µm³
- Standard dev: 9,283,381.71 µm³

#1_4a-2, ROI 1 – Mean Feret Diameter
Omphacite 1

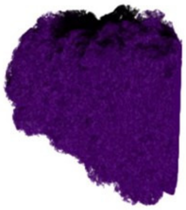

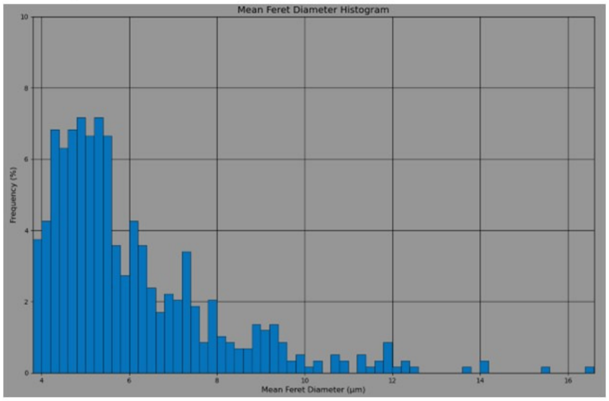

Mean Feret Diameter Histogram statistics

- Min: 3.80 µm
- Max: 560.83 µm
- Mean: 7.19 µm
- Standard dev: 22.75 µm

#1_4a-2, ROI 1 – Volume
Omphacite 1

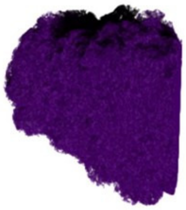

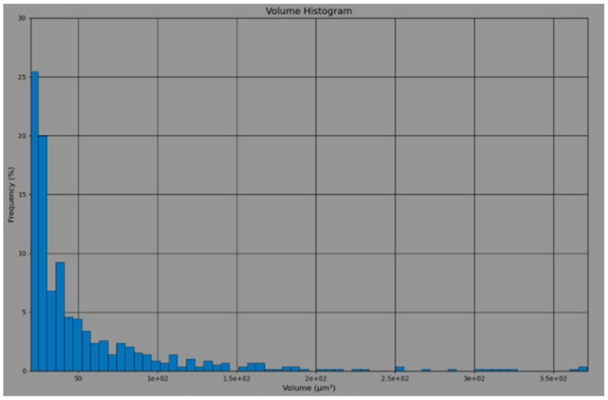

Volume Histogram statistics
- Min: 19.08 µm³
- Max: 19,564,034.74 µm³
- Mean: 32,671.39 µm³
- Standard dev: 798,029.88 µm³

#1_4a-2, ROI 1 – Mean Feret Diameter
Garnet

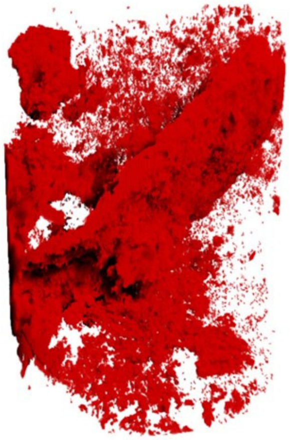

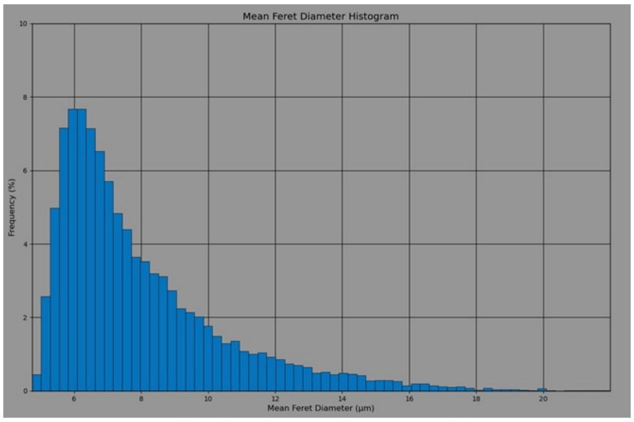

Mean Feret Diameter Histogram statistics
- Min: 4.74 µm
- Max: 959.59 µm
- Mean: 8.84 µm
- Standard dev: 10.24 µm

#1_4a-2, ROI 1 – Volume
Garnet

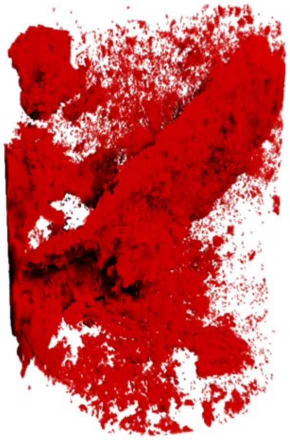

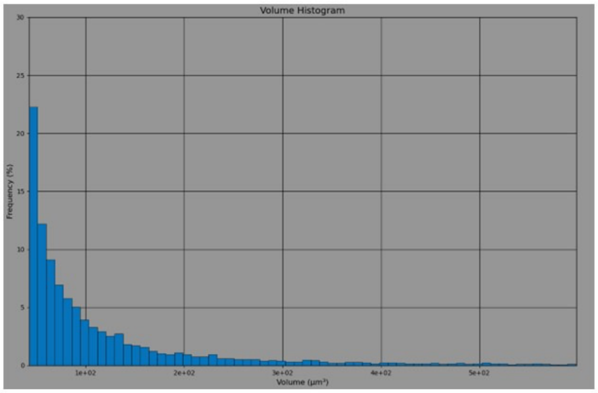

Volume Histogram statistics
- Min: 42.40 µm³
- Max: 61,702,783.01 µm³
- Mean: 5,343.29 µm³
- Standard dev: 538,276.49 µm³

#1_4a-2, ROI 1 – Mean Feret Diameter
Titanite

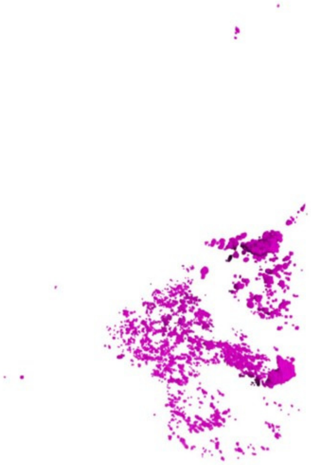

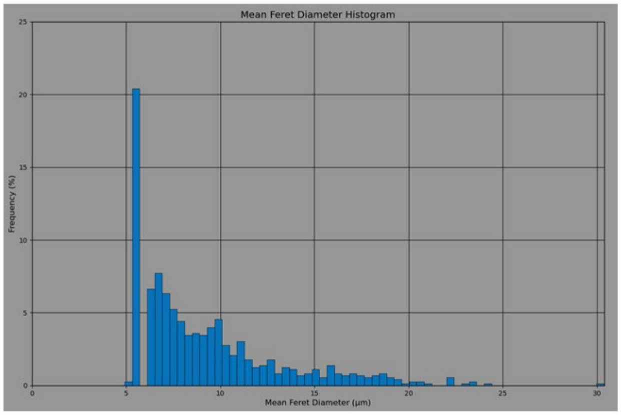

Mean Feret Diameter Histogram statistics
- Min: 4.92 µm
- Max: 139.49 µm
- Mean: 11.35 µm
- Standard dev: 9.94 µm

#1_4a-2, ROI 1 – Volume
Titanite

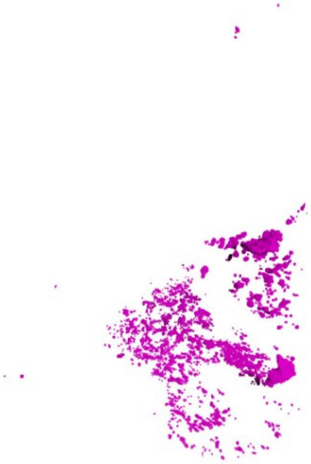

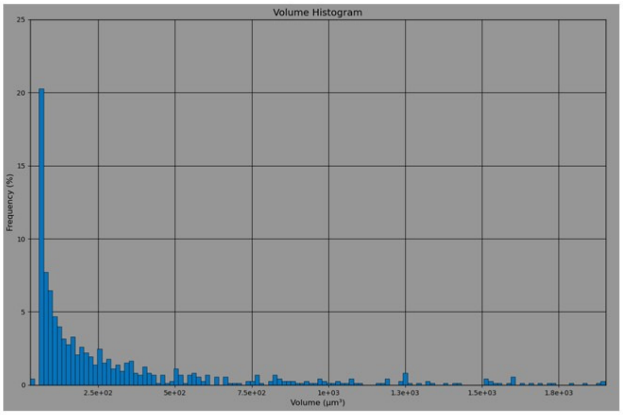

Volume Histogram statistics

- Min: 27.56 µm³
- Max: 110,334.97 µm³
- Mean: 1,035.32 µm³
- Standard dev: 6,132.24 µm³

#1_4a-2_ROI 2

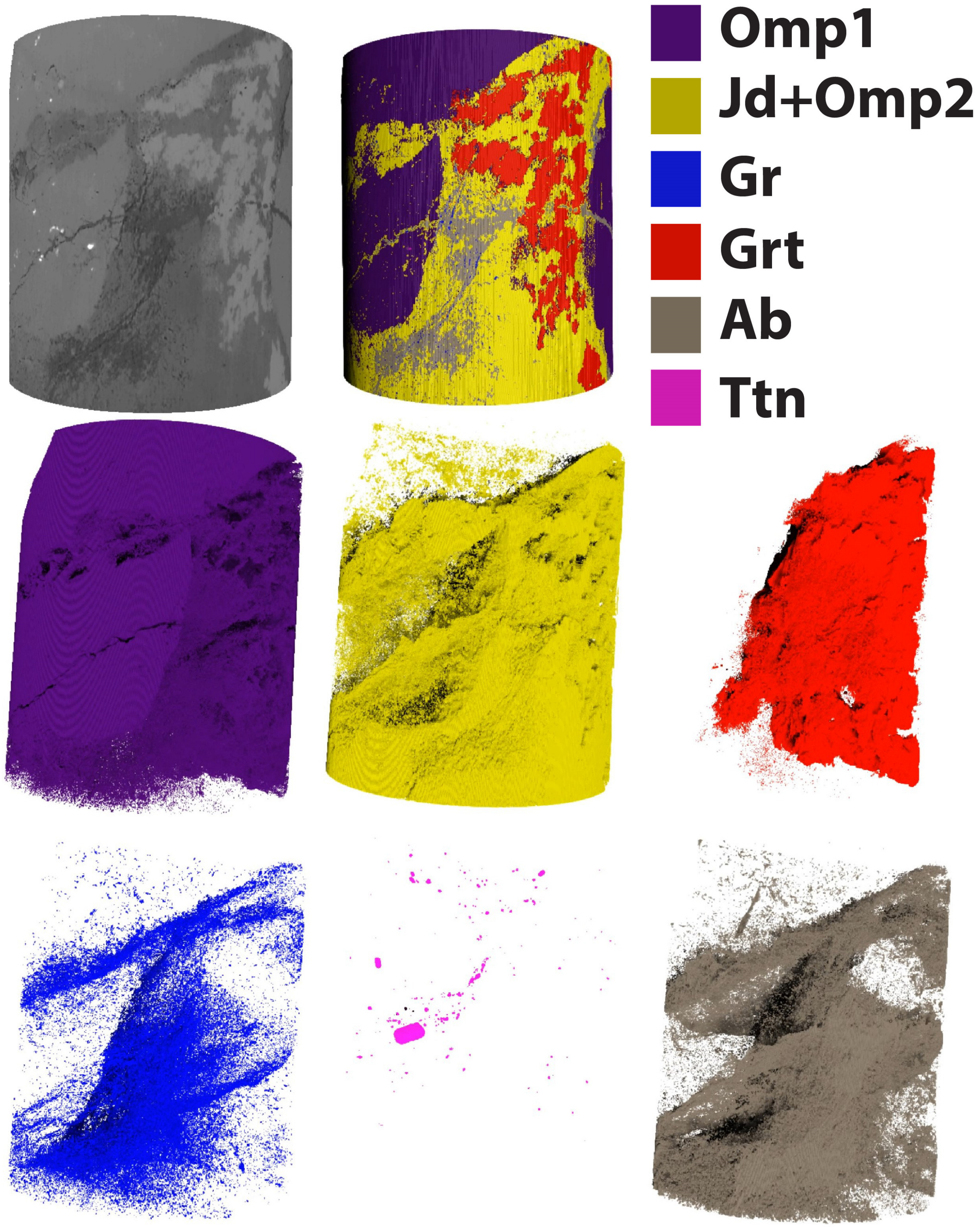

#1_4a-2, ROI 2

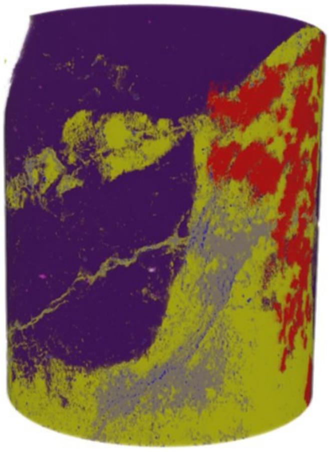

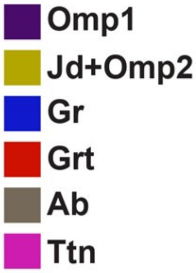

Titanite	0.06 %
Garnet	11.85 %
Graphite	0.66 %
Albite	8.11 %
Onfacite 2 & Jadeite	23.22 %
Omhpacite 1	55.32 %

Sum	99.22 %

#1_4a-2, ROI 2 – Volume Graphite

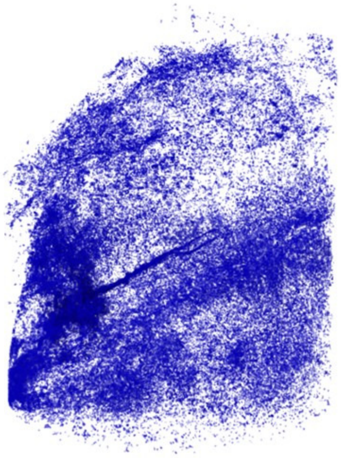

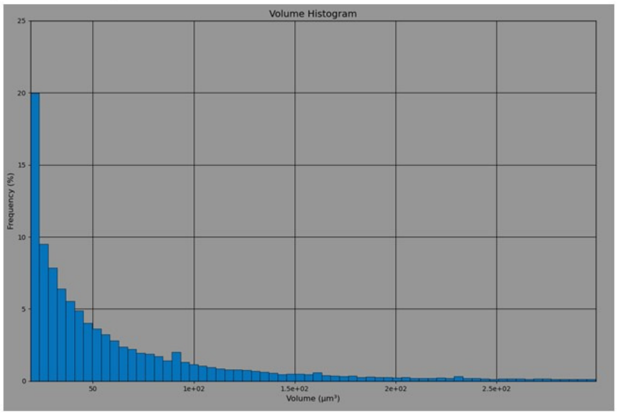

Volume Histogram statistics

- Min: 19.08 µm³
- Max: 1,123,805.53 µm³
- Mean: 142.87 µm³
- Standard dev: 5,762.18 µm³

#1_4a-2, ROI 2 – Mean Feret Diameter
Graphite

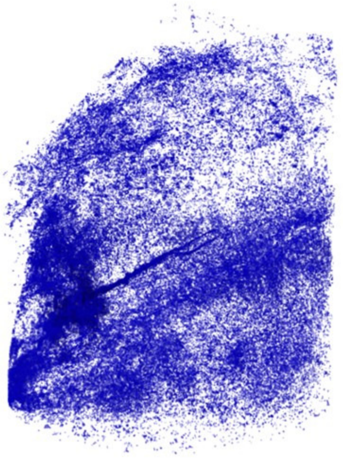

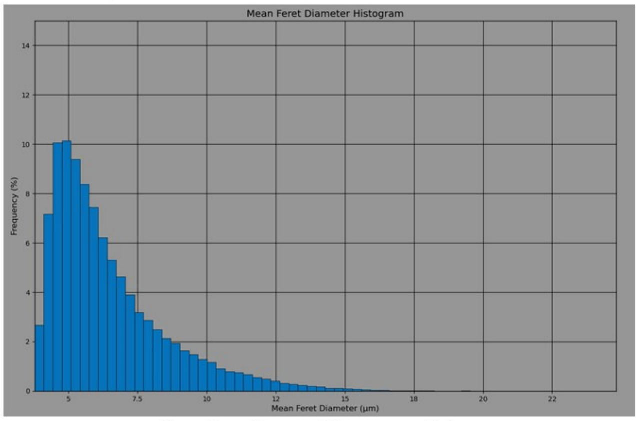

Mean Feret Diameter Histogram statistics
- Min: 3.77 µm
- Max: 435.07 µm
- Mean: 7.05 µm
- Standard dev: 4.72 µm

#1_4a-2, ROI 2 – Volume
Albite

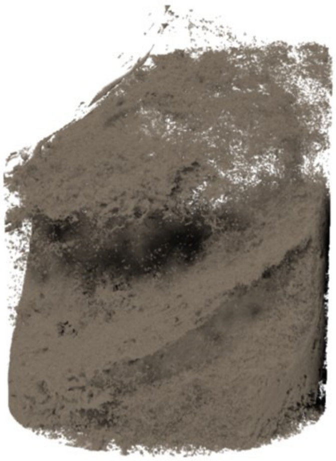

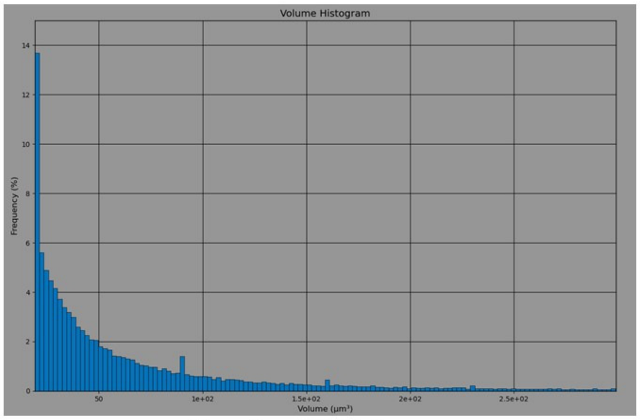

Volume Histogram statistics
- Min: 19.08 µm³
- Max: 72,430,369.72 µm³
- Mean: 1,417.70 µm³
- Standard dev: 304,958.97 µm³

#1_4a-2, ROI 2 – Mean Feret Diameter
Albite

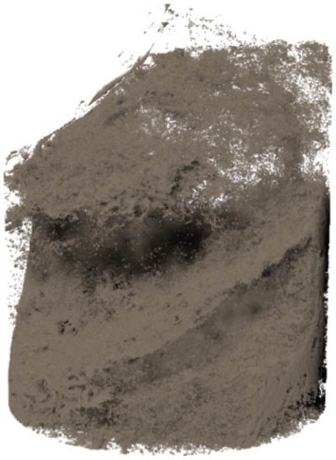

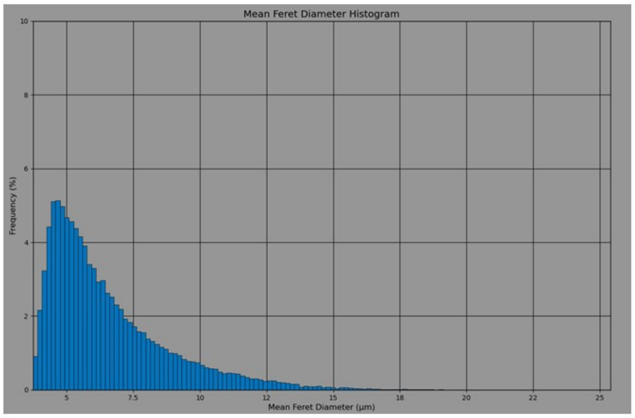

Mean Feret Diameter Histogram statistics

- Min: 3.73 µm
- Max: 1,201.34 µm
- Mean: 7.40 µm
- Standard dev: 7.02 µm

#1_4a-2, ROI 2 – Volume
Omphacite 2 & Jadeite

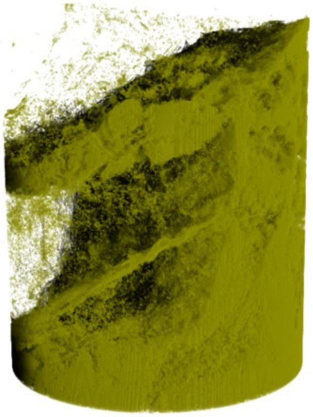

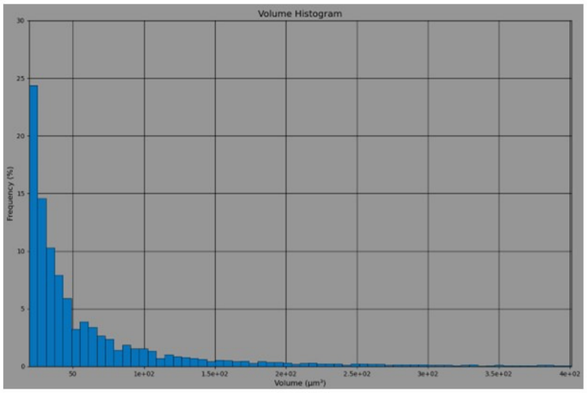

Volume Histogram statistics

- Min: 19.08 µm³
- Max: 225,620,921.56 µm³
- Mean: 6,578.16 µm³
- Standard dev: 1,208,846.91 µm³

#1_4a-2, ROI 2 – Mean Feret Diameter
Omphacite 2 & Jadeite

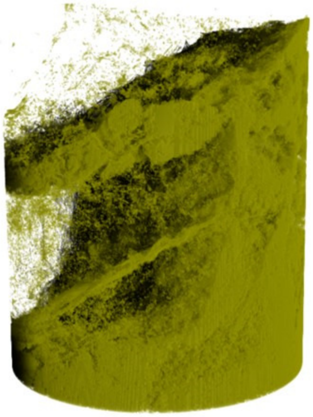

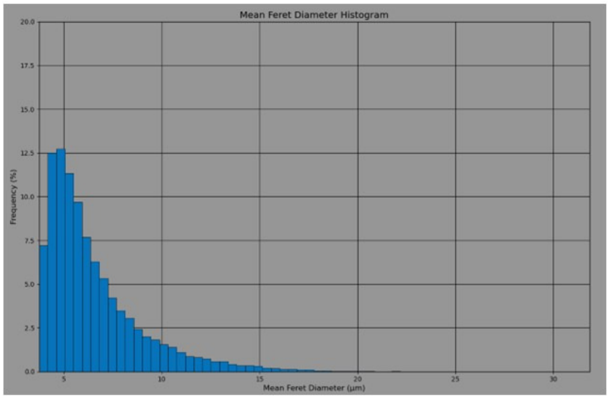

Mean Feret Diameter Histogram statistics

- Min: 3.74 µm
- Max: 1,256.09 µm
- Mean: 7.10 µm
- Standard dev: 7.94 µm

#1_4a-2, ROI 2 – Volume
Ompacite 1

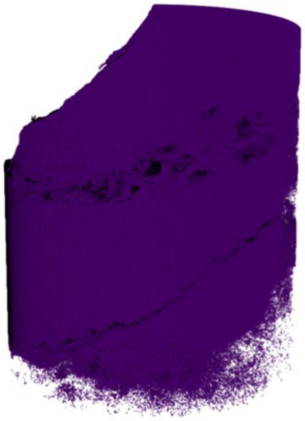

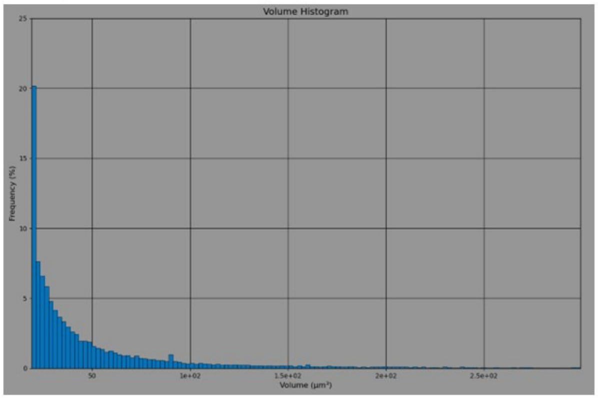

Volume Histogram statistics

- Min: 19.08 µm³
- Max: 541,655,817.03 µm³
- Mean: 15,466.43 µm³
- Standard dev: 2,886,296.94 µm³

#1_4a-2, ROI 2 – Mean Feret Diameter
Omphacite 1

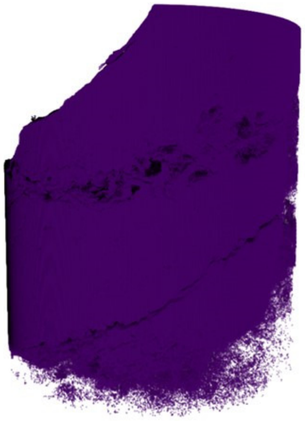

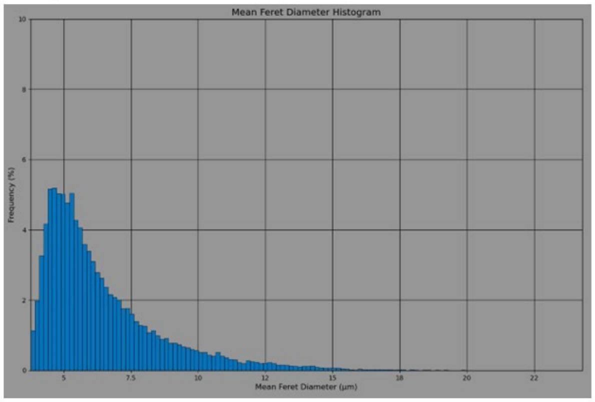

Mean Feret Diameter Histogram statistics

- Min: 3.77 µm
- Max: 1,351.93 µm
- Mean: 6.95 µm
- Standard dev: 8.20 µm

#1_4a-2, ROI 2 – Volume
Garnet

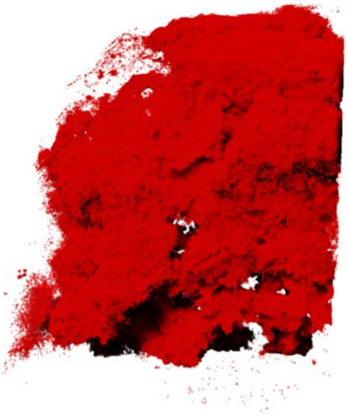

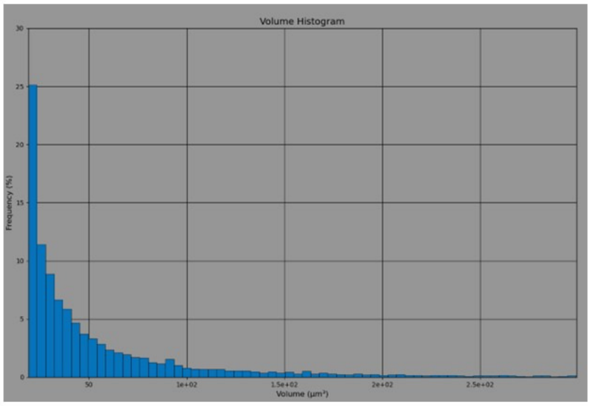

Volume Histogram statistics

- Min: 19.08 µm³
- Max: 115,643,919.91 µm³
- Mean: 9,252.11 µm³
- Standard dev: 1,029,502.13 µm³

#1_4a-2, ROI 2 – Mean Feret Diameter
Garnet

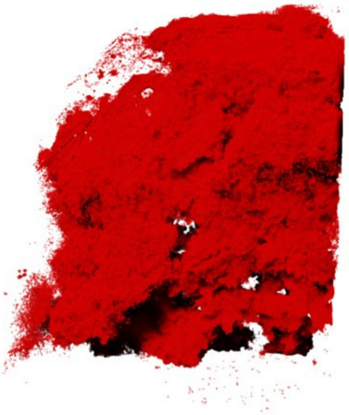

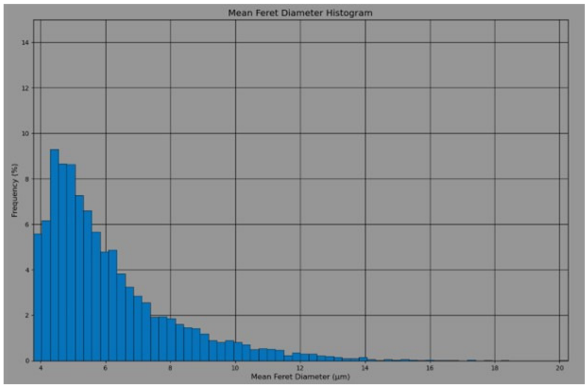

Mean Feret Diameter Histogram statistics
- Min: 3.78 µm
- Max: 998.49 µm
- Mean: 6.60 µm
- Standard dev: 9.43 µm

#1_4a-2, ROI 2 – Volume
Titanite

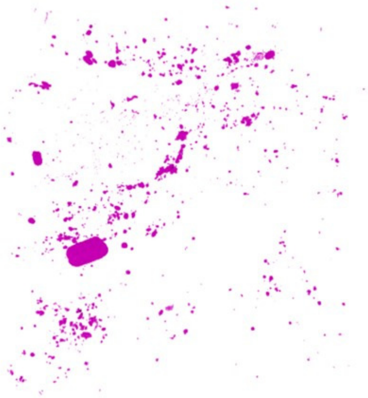

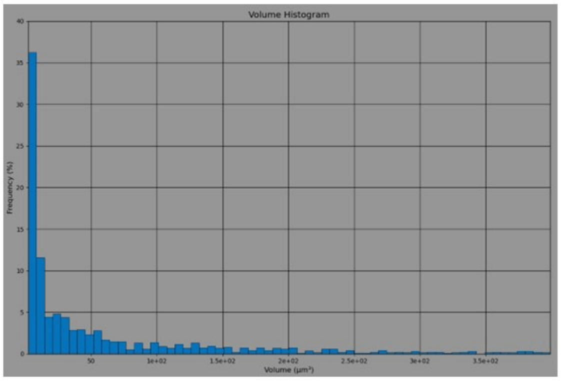

Volume Histogram statistics
- Min: 2.12 µm³
- Max: 261,452.23 µm³
- Mean: 365.01 µm³
- Standard dev: 6,763.20 µm³

#1_4a-2, ROI 2 – Mean Feret Diameter
Titanite

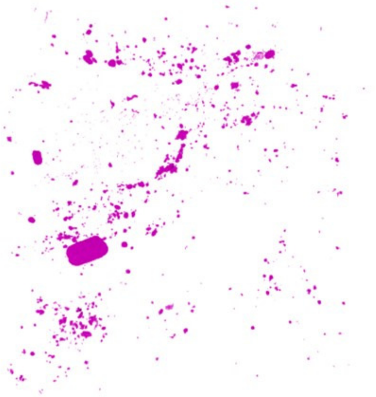

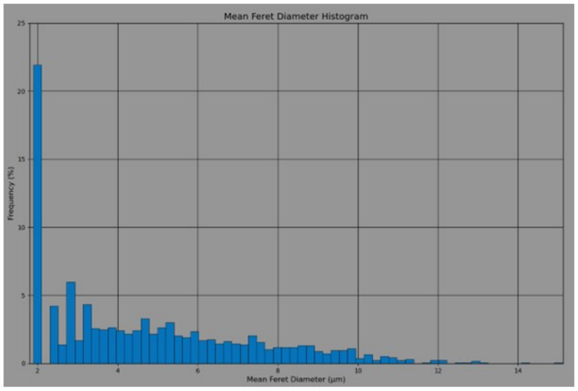

Mean Feret Diameter Histogram statistics

- Min: 1.89 µm
- Max: 96.62 µm
- Mean: 5.96 µm
- Standard dev: 5.44 µm
