# Supplementary material for: Methane-hydrogen-rich fluid migration may trigger seismic failure in subduction zones at forearc depths
Source: Nat Commun. 2024 Jan 11;15:480. doi: 10.1038/s41467-023-44641-w (PMC10784519; doi:10.1038/s41467-023-44641-w)
Supplement: Supplementary file 3 — Description of Additional Supplementary Files [file 41467_2023_44641_MOESM3_ESM.pdf]

Description of Additional Supplementary Files

Supplementary Movie 1. X-ray Microscopy Video of sample #1_4a-2 showing the distribution of mineral phases appearing one after another.

Supplementary Movie 2. X-ray Microscopy Video of sample #1_4a-2 showing the distribution of mineral phases appearing one mineral at a time.

Supplementary Movie 3. X-ray Microscopy Video of sample #1_4a-2 regions of interest

1 showing the distribution of mineral phases appearing one after another.

Supplementary Movie 4. X-ray Microscopy Video of sample #1_4a-2 regions of interest

1 showing the distribution of mineral phases appearing one mineral at a time.

Supplementary Movie 5. X-ray Microscopy Video of sample #1_4a-2 regions of interest

2 showing the distribution of mineral phases appearing one after another.

Supplementary Movie 6. X-ray Microscopy Video of sample #1_4a-2 regions of interest 2 showing the distribution of mineral phases appearing one mineral at a time.
